# Memory from nonsense syllables to novels: A survey of retention

**DOI:** 10.3758/s13423-024-02514-3

**Published:** 2024-05-07

**Authors:** Gabriel A. Radvansky, Dani Parra, Abigail C. Doolen

**Affiliations:** Department of Psychology, University of Notre Dame, 390 Corbett Hall, Notre Dame, IN 46556 USA

**Keywords:** Memory, Forgetting, Loss functions

## Abstract

Memory has been the subject of scientific study for nearly 150 years. Because a broad range of studies have been done, we can now assess how effective memory is for a range of materials, from simple nonsense syllables to complex materials such as novels. Moreover, we can assess memory effectiveness for a variety of durations, anywhere from a few seconds up to decades later. Our aim here is to assess a range of factors that contribute to the patterns of retention and forgetting under various circumstances. This was done by taking a meta-analytic approach that assesses performance across a broad assortment of studies. Specifically, we assessed memory across 256 papers, involving 916 data sets (e.g., experiments and conditions). The results revealed that exponential-power, logarithmic, and linear functions best captured the widest range of data compared with power and hyperbolic-power functions. Given previous research on this topic, it was surprising that the power function was not the best-fitting function most often. Contrary to what would be expected, a substantial amount of data also revealed either stable memory over time or improvement. These findings can be used to improve our ability to model and predict the amount of information retained in memory. In addition, this analysis of a large set of memory data provides a foundation for expanding behavioral and neuroimaging research to better target areas of study that can inform the effectiveness of memory.

**Supplementary Information:**

The online version contains supplementary material available at 10.3758/s13423-024-02514-3.

An important aim of cognitive psychology is to understand the progress of memories over time (Ebbinghaus, 1885). One issue that memory researchers have struggled with for well over a century is how much is remembered and forgotten as time passes. This is important because understanding the progress of memory would allow us to predict memories at different points in the future. Ebbinghaus (1885) proposed a logarithmic function to capture memory retention over time. Other researchers have suggested other functions, including power (e.g., J. R. Anderson & Schooler, 1991; Averell & Heathcote, 2011; Rubin & Wenzel, 1996a, 1996b; Wickelgren, 1974; Wixted & Carpenter, 2007; Wixted & Ebbesen, 1991a, 1991b), exponential (e.g., Loftus, 1985a, 1985b), and linear functions (Fisher & Radvansky, 2019). The aim of this study was to analyze a very large corpus of data sets from well over a century of memory research to better understand which pattern(s) best captures the progress of memory retention and forgetting over time, and to assess whether the type of pattern observed may be influenced by other factors, such as the retention delay, the type of materials tested, the memory test used, and so on.

An earlier attempt at this was reported by Rubin and Wenzel (1996a, 1996b). They assessed how well 210 published data sets fit 105 two-parameter functions. Their criteria for data inclusion were that (a) there were five or more retention intervals, (b) the dependent measure conveyed how much was remembered, and (c) each included data set fit at least one of their 105 two-parameter functions with an *r*^*2*^ of .90 or better. Their assessment largely involved counting the number of times each of the 105 functions was one of the ten best for a given data set. These two-parameter functions were selected because they have (a) a measure of the rate of change over time and (b) a scaling measure. Using this approach, Rubin and Wenzel concluded that the four best-fitting two-parameter functions were (a) logarithmic, (b) power, (c) exponential in the square root of time (exponential-power), and (d) hyperbolic in the square root of time (hyperbolic-power).

Rubin and Wenzel (1996a, 1996b) classified and sorted the data based on the labs that the data came from (e.g., Wickelgren and Bahrick) and study types (e.g., autobiographical memory). Our study expands their assessment by considering the amount of data in a study (e.g., number of participants and observations). Our aim was to build upon the impressive effort of Rubin and Wenzel to systematically assess and compare different retention and forgetting patterns for various independent and dependent variables, such as retention delay and memory test types, and to examine why the pattern of retention and forgetting might vary as a function of such factors.

Like Rubin and Wenzel (1996a, 1996b), we take a largely atheoretical, exploratory approach to our analyses. That said, we do consider some theoretical issues that our analysis can inform and adjudicate. This assessment of patterns of retention and forgetting is motivated by recent work in our lab. One line of work showed that there are changes in the rate of forgetting for different periods of time (Radvansky et al., 2017a, 2017b, 2022). Another reports that some patterns of retention and forgetting do not conform to a negatively accelerating pattern but are better captured by a linear function (Fisher & Radvansky, 2019). This is important because with a negatively accelerating function, there is often a *constant proportional* loss over some unit of time (e.g., log time for a power function). In contrast, for a linear function there is a *constant amount* of loss over a given unit of time. Thus, there would be an increasing proportion of information lost with longer and longer periods of time.

For this study, we added more recent memory studies and used somewhat different criteria than Rubin and Wenzel (1996a, 1996b). This larger dataset allows for a more systematic assessment of a range of variables that may contribute to the observed memory performance. In what follows, we detail the inclusion criteria as well as the independent and dependent variables used for our analysis. Our analysis is composed of two phases. We first assess the goodness-of-fit of various functions to see which function(s) emerge as best descriptors of memory, similar to the procedure used by Rubin and Wenzel (1996a, 1996b). We then assess various factors that contribute to the emergence of those functions.

## Memory functions

Our assessment of memory loss was done for five target functions (Table 1): logarithmic, power, hyperbolic-power, exponential-power, and linear. These are all two-parameter^1^ functions in which memory, *M*, across time, *t*, is captured by a constant scale parameter, *a*, and a rate of change, *b.* Each of these functions is now considered in turn.

**Table 1:** Function equations considered in our analyses

Function	Equation
Logarithmic	M = a – b*ln(t)
Power	*M = at*^*b*^
Exponential-power	*M = ae*^-b*√t^
Hyperbolic-power	*M = 1/(a + b*√t)
Linear	M = bt + a

### Logarithmic

Logarithmic functions have been attributed to Ebbinghaus^2^ (1885; see also Woodworth, 1938) and were among the best-fitting functions in Rubin and Wenzel’s (1996a, 1996b) assessment. They suggested that these functions may be best for non-autobiographical memories. For this function, a boundary condition is necessary. Logarithmic loss functions predict that at some point people will remember less than nothing, which is nonsensical. Thus, there should be a constraint restricting values to be positive for the appropriate application of logarithmic functions to memory data.

### Power

Power functions were also among the best-fitting functions reported by Rubin and Wenzel (1996a, 1996b) and are often preferred by many researchers studying patterns of retention and forgetting (e.g., Wixted & Ebbesen, 1991a, 1991b). As noted by Rubin and Wenzel, with a power function, there are equal ratios of retention time that are accompanied by unequal ratios of memory retrieval. It has been suggested that power function occur as a result of memory consolidation processes (Wixted, 2004a, 2004b) or to mirror the occurrence of events in the environment (Anderson & Schooler, 1991).

### Exponential-power

The exponential in the square root of time, which is a special case of the Weibull function, was first suggested as a description of memory change by Wickelgren (1972). This function was also among the best-fitting functions reported by Rubin and Wenzel (1996a, 1996b).

### Hyperbolic-power

The fourth function that did well in Rubin and Wenzel’s (1996a, 1996b) analysis was the hyperbola in the square root of time. However, it is rarely considered as a means of capturing data from human participants outside of their study. It has, as suggested by Rubin and Wenzel, been more popular in research with animals (e.g., Harnett et al., 1984a, 1984b; Staddon, 1983a, 1983b).

### Linear

The last function that we consider is the linear function. Although it did not make Rubin and Wenzel’s (1996a, 1996b) top four list, recent work suggests that it may be observed under some circumstances (Fisher & Radvansky, 2019). Thus, we include it here to better understand the circumstances under which it would appear. Like logarithmic functions, linear functions also predict that at some point, memory becomes negative. Thus, these functions also need to be used with a boundary constraint so that they cannot drop below zero. That said, it has been suggested that a linear forgetting pattern may be reflective of curvilinear patterns “by assuming that it simply reflects a scaling/measurement artifact” (Wixted, 2022, p. 1785), and as such, linear patterns of forgetting could easily be dismissed.

These five functions describe memory loss over time. We also consider cases in which memory remains *stable* over extended retention intervals. For our purposes, we define data sets as falling into this category when the net proportion change across a data set’s retention intervals is between −.01 and .01. Although these data sets may not contribute to understanding the function of best fit (because the data may be so flat), it is useful to understand the situations in which stable memory arises. Following this logic, we also separately consider cases in which memory *increases* over time. Again, examining such cases helps identify the circumstances under which improvement rather than loss might be observed with the aim of contributing to a more robust understanding of memory retention.

Previous reviews of retention and forgetting, such as Rubin and Wenzel’s (1996a, 1996b) classic assessment, have concluded that different functions capture different patterns of loss that have been observed in the literature. Different retention and forgetting functions are likely to occur because different memory representations and processes are involved, leading to the observation of different functions. These can then be used to better explore and understand how memory retention, loss, and retrieval operate. As one example, Fisher and Radvansky (2022a, 2022b) found that less well-known information was better captured by a power function, whereas better-learned information was better captured by a linear function. This was explained using a computational model of memory retrieval, the RAFT model. In brief, the explanation is that better-learned information allows for more reconstruction of partial knowledge in memory, which can produce a linear forgetting function, even if the individual elements making up a representation are lost in a way that follows a power function. This is similar to the idea that the more components of an event that can be used as retrieval cues, the better memory performance will be (Jones, 1976). In comparison, with less well-learned information, reconstruction is more difficult, and a power function emerges more readily. Thus, with knowledge such as this, knowing which function best fits the data can provide some understanding of how information is represented and processed in memory.

## Inclusion criteria

There are a number of criteria for each data set to meet to be included in our corpus and analyses. These are listed in Table 2 and are described here, along with a justification for each.

**Table 2:** Inclusion criteria for data sets to be included in our corpus

Study characteristics
Published in or translated into English
Three or more retention intervals
Measurement of memory
Sample characteristics
Human participants
Adults aged 18 or older
No psychopathology
No altered cognitive state

### Study characteristics

#### English

We limited our search to studies that are either published in or translated into English to ensure an accurate understanding of the methods and results of each study. The one exception to this is a study by Radosavljevich (1907a, 1907b), which is included here because of its historical significance.

#### Number of retention intervals

For our analyses, we look at memory retention and forgetting over time, so the studies needed to measure memory after different retention delays. We used studies with three or more retention intervals because three data points are needed to fit the two-parameter functions currently considered. Rubin and Wenzel (1996a, 1996b) limited themselves to studies with five or more retention intervals to increase data stability. While including studies with three or four retention intervals does introduce some potential instability, we include them to increase the size of our corpus. The number of retention intervals is included as a factor in the analyses to account for any potential influences of it.

#### Measurement

Each included data set had a clear measure of the *amount* of information retained in memory, such as the amount recalled, recognized, or degree of savings. For our analyses this was uniformly represented as proportion correct.^3^ We further restrict ourselves to studies in which declarative memory was measured. Procedural memory contains information that cannot be consciously recalled, such as how to ride a bicycle, and is thought to involve different neural mechanisms (e.g., Cohen et al., 1985; Squire, 1986). Moreover, it is much less clear how to quantify the proportion remembered for such data. Thus, studies of procedural memory retention were excluded.

We also exclude studies in which people made free memory associations to individual words, and the data are based on what people produced initially (e.g., Crovitz & Shiffman, 1974; Rubin & Wenzel, 1996a, 1996b). With this method, there is no targeted assessment of memories at different times. These were simply the first memories that come to mind when people hear those words. This is not an assessment of the proportion of memories accessible at different time periods. Thus, we do not know how much people do and do not remember from different periods of time.

### Sample characteristics

For our analyses, we include studies using human adults, with no known psychopathologies.

#### Humans

Unlike Rubin and Wenzel (1996a, 1996b), who included animal studies, we confine our analyses to data sets that involve humans. Memory research with non-humans has been invaluable, as there are many points of convergence between the two (e.g., Squire, 2004). However, there are also notable dissimilarities that could complicate our analyses (e.g., Premack, 2007).

#### Age

We limit our analyses to data that do not involve children (younger than 18). There are both neurological developments and behavioral changes that occur throughout childhood that could complicate our analyses. Thus, we chose to be conservative and not include data from children here.

#### Psychopathology and cognitive state

We excluded samples that assessed people with a known psychopathology, such as amnesia or schizophrenia. We also excluded samples in which the participant’s cognitive state was altered by a substance such as alcohol or caffeine. Again, memory in these groups varies from normal (e.g., Aleman et al., 1999; Wickelgren, 1975). If there was a neurotypical control group in a study, we did include that data.

### A note on fit characteristics and data reduction

Our analyses examine the influence of several factors on the goodness-of-fit, as indicated by the coefficient of determination, *r*^2^, of several functions to the retention data of each study. Rubin and Wenzel (1996a, 1996b) used a strict criterion of *r*^2^ ≥ .90 for at least one of their functions to focus on well-behaved data sets. We elected not to use this criterion because, although the fit of individual studies may not be as high, the various factors included in our analysis may account for such variance.

However, because our emphasis is on assessing which function(s) best fit the data, we removed any data sets that were so variable that none of the functions captured well the nature of changes in performance. After assembling our corpus, we took some steps to reduce the amount of data used. We first dropped any data sets, in cases of memory loss, in which the fit of any of the five functions was poor. Rather than dropping anything below *r*^2^ = .90, as Rubin and Wenzel (1996a, 1996b) had done, we took a more inclusive approach. We instead took the best-fitting *r*^2^ for each study, across the five retention functions, in addition to keeping any data showing no net change or improvement. We then chose to drop those data sets for which the best-fitting function was less than *r*^2^ = .5. This is around two standard deviations from the mean of the five best-fitting functions (*M* = .880, *SD* = .192), again excluding no net change and improving data sets. This resulted in the loss of 53 data sets, which had a low average best fit (*M* = .31, *SD* = .14). The reduced data set used for our analyses can be found in our online Supplement A (all supplements are available at https://osf.io/wq9ty). For interested readers, the removed data are provided in our online Supplement B. After dropping the poorly-fitting data, the data sets showing memory loss over time were better described by the five mathematical functions (*M* = .922, *SD* = .112).

In terms of prominent differences, relative to the retained data sets, the sets that were removed had slightly smaller average sample sizes (*M* = 98.2 vs. *M* = 110.0), smaller average groups sizes (*M* = 24.3 vs. *M* = 30.3), and fewer observations per person (*M* = 44.3 vs. *M* = 120.1). They also had a higher proportion of multiple study opportunity data sets (*M* = .71 vs. *M* = .44). Moreover, the trimmed data had more retention intervals (*M* = 5.5 and *M* = 4.7) and covered longer average periods of time (*M* = 18.7 years and *M* = 8.3 years). The trimmed data also had an average lower initial memory (*M* = .65 and *M* = .73), and importantly, the trimmed data sets were more likely to involve less change from one retention interval to the next (*M* = −.02 vs. *M* = −.07). Thus, it seems likely that the data sets that were trimmed out had poor retention function fits because they had less data overall, for information that was less well-learned, over longer periods of time, and were less likely to show much change over time.

### Literature search

Many studies of memory with multiple retention intervals are not labeled as such, so it is far too limiting to do a literature search merely using key terms. We have found published work examining forgetting over time (e.g., Rubin & Wenzel, 1996a, 1996b) and have sifted through the references of each to find additional papers. We also included any papers and data sets that we have happened upon during this process. We hope that the reports that are included in this analysis are representative of the population of studies that fit our inclusion criteria. From this set of criteria, we have developed our corpus. This includes data from 256 papers, involving 916 data sets (e.g., multiple experiments and/or conditions within articles).^4^ That said, this corpus is almost certainly incomplete.

## Data coding

There were a range of variables considered for our analyses (Table 3). These are about general characteristics of a study, characteristics of the materials, learning characteristics, nature of the retrieval tasks, and aspects of retention. The summary statistics for these variables are provided in Table 4. The data used here as well as the syntax for analysis are publicly available on the Open Science Framework (https://osf.io/wq9ty).

**Table 3:** Independent variables considered in our analyses

Variable	Type
General characteristics	
Publication year	Numeric
Sample size	Numeric
Material characteristics	
Material type	Categorical
Material complexity	Ordinal
Learning characteristics	
Multiple study opportunities	Numeric
Degree of learning	Numeric
Distractor task	Categorical
Test characteristics	
Assessment type	Categorical
Number of retention intervals	Numeric
Study design	Categorical
Number of observations per person	Numeric
Overall amount of data	Numeric
Retention characteristics	
Shortest retention Interval	Numeric
Longest retention Interval	Numeric
Average retention Interval	Numeric
Retention range (longest–shortest)	Numeric
Initial memory	Numeric

**Table 4:** Summary statistics of the numeric independent variables for our corpus

Independent variable	Mean	Median	Low	High	*SE*
Publication year	1993.9	1998	1885	2022	.77
Sample size	109.4	26.5	1	4,239	9.4
Observations per participant	115.8	32	1	15,964	25.4
Amount of data	3506.9	1120	10	127,170	310.8
Number of retention intervals	4.7	4	3	52	.10
Shortest retention interval (in seconds)	35,352,101 (1.11 years)	300 (5 min.)	.01	630,720,000 (20 years)	2,938,141 (1.1 mon.)
Longest retention interval (in seconds)	279,984,551 (8.9 years)	1,814,400 (3 weeks)	5.9	2,144,448,000 (68 years)	18,005,327 (6.9 mon.)
Average retention interval (in seconds)	148,179,639 (4.7 years)	605,413 (1 week)	3.5	1,492,704,000 (47 years)	9,805,150 (3.8 mon.)
Retention range (in seconds)	244,632,450 (7.8 years)	1,814,370 (3 weeks)	4.4	1,860,624,000 (59 years)	15,814,291 (6.1 mon.)
Initial memory	.73	.79	.027	1.00	.008

For ease of understanding: 1 minute = 60 seconds, 1 hour = 3,600 seconds, 1 day = 8,640 seconds, 1 week = 604,800 seconds, 1 month (30 days) = 2,592,000 seconds, 1 year = 31,536,000 seconds.

### General characteristics

#### Publication year

Research practices in the study of human memory have changed over the decades. These changes may contribute to the observed data in ways that are not captured by our other factors. To allow for this, we include the year of publication as a dependent variable. The description of the various years in our corpus is provided in Table 4.

#### Sample size/observations per participant/amount of data

All else being equal, studies with larger sample size, more observations per participant, and more data in general, are likely to have more stable data that more accurately reflect the population (Cohen, 1992a, 1992b; Cronbach et al., 1972; Marcoulides, 1993; VanVoorhis & Morgan, 2007). Studies with fewer data points may overestimate true effect sizes and, as such, may be less replicable (e.g., Button et al., 2013). Specifically, if certain functions are better fit by small data sets, this would bring into question the accuracy of such functions. The description of the sample sizes, observations per participant, and amount of data in our data set is provided in Table 4.

### Material characteristics

#### Material type

There are different types of memory for different types of information (e.g., nonsense syllables and novels). Thus, we also coded for the types of materials used. We identified many different material types. These are listed in Table 5.

**Table 5:** Levels of material complexity used for our assessment

Level	Materials
1	letters, characters, letter trigrams, letter strings, nonsense syllables, visual arrays, abstract images
2	words, idioms, class grades, names, faces, pictures of objects, odors
3	word–digit pairs, symbol–digit pairs, word–trigram pairs, English–Swahili pairs, spatial position
4	word pairs, word triads generated words, word definitions, new vocabulary words, math problems, famous faces, famous names, famous voices, names of television programs, names of racehorses
5	sentences, classroom concepts, word generation, pictures of scenes, famous scenes
6	poems, a directed walk through town, faces and events, events and names
7	stories, course material, autobiographical events, flashbulb events, public news events, novels, videos of activities

#### Material complexity

The level of complexity of the material was coded to convey the degree to which the materials likely activate prior world knowledge and invite inferences. The levels of complexity are shown alongside the material types in Table 5. Level 1 (*n* = 124 data sets) includes materials that have very little to no semantic meaning, such as nonsense syllables, and are presented in isolation with no reference to other items. Level 2 (*n* = 252) includes materials that have semantic meaning, either through prior knowledge or individual experiences. However, the items are presented in isolation with little to no relation to one another. Thus, while elaboration is possible, it is likely to be limited in scope.

Level 3 (*n* = 37) includes materials in which there is some type of association. However, while one of the items may be meaningful, the other is not, at least from the perspective of the participant. Again, while elaboration is possible, it is likely to be limited. Level 4 (*n* = 145) includes materials for which there is an association between two or more meaningful items, however, not enough to form a complete proposition. Some elaboration might be possible, but it would likely be subjective and initiated by the person.

Level 5 (*n* = 91) includes materials that convey at least one complete idea or proposition. These materials are much more likely to encourage some elaborative processing. Level 6 (*n* = 24) includes materials that clearly go beyond a single propositional idea unit and involve multiple ideas. Thus, there is more opportunities for elaborative processing. However, more complex information is less likely to convey a coherent situation or event, or a collection of situations or events. The faces and events material types are placed here because they involve elements of simpler and more complex materials. Poems are placed here because they typically do not convey elaborative descriptions of events as prose does. Level 7 (*n* = 243) includes materials that clearly involve an understanding of situations and events, that often span across time, with many different elements and inter-relations, such as novels. Given recent findings of linear forgetting in complex materials, it is expected that material complexity may be related to linear function fit (Fisher & Radvansky, 2019).

Given that our complexity measure provides an index that can be more readily used to compare with other measures, and the different memoranda were sorted into the seven complexity classifications, we elected not to use any coding of the specific memoranda in any of our analyses. This information is available for any readers wishing to explore such issues.

### Learning characteristics

#### Multiple study opportunities

Memory can vary depending on whether information was presented once, or if there were multiple study opportunities (e.g., Ebbinghaus, 1885). This was coded in our corpus as 0 for single (*n* = 471 data sets) and 1 for multiple (*n* = 445).

#### Degree of learning

Another factor that can influence the consolidation of information into memory is the degree of learning. The better the information is learned, the more likely that it has been stored in memory (e.g., Craik & Lockhart, 1972). This could have consequences for the nature of the pattern of retention and forgetting that is observed. To quantify this for the purposes of our analyses, we came up with a rough measure to code these studies by identifying four levels. Level 1 are materials from Complexity Levels 1 to 4 that likely involved rote rehearsal and were only explicitly processed once (*n* = 310). Level 2 are materials from Levels 1 to 4 and were explicitly processed more than once (*n* = 248). Level 3 are materials from Complexity Levels 5 to 7 that likely involved elaborative rehearsal and were explicitly processed once (*n* = 178). Finally, Level 4 are materials from Levels 5 to 7 that were explicitly processed more than once (*n* = 180).

#### Distractor task

Studies vary in whether there was an experimenter-imposed distractor task used to encourage forgetting. Memory retention requires that traces go through a process of consolidation (e.g., McGaugh, 1966, 2000). Prior to this, traces might be disrupted through distraction, leading to faster forgetting. Thus, it is possible that the pattern of forgetting would differ in studies in which a distractor task was present (*n* = 150) versus those in which it was not (*n* = 766).

### Test characteristics

#### Assessment type

This is the type of memory test used. In our data set, these include free recall (*n* = 432 data sets), cued recall (*n* = 121), yes–no recognition (*n* = 141), forced choice recognition (*n* = 160), savings (*n* = 14), stem completion (*n* = 22), fragment completion (*n* = 12), anagram solution (*n* = 1), matching (*n* = 7), problem solutions (*n* = 4), and source monitoring (*n* = 2).

#### Number of retention intervals

We coded for the number of retention intervals in each data set. Data with more retention intervals may have more stable patterns than data with fewer, and this needs to be considered. This variable allows us to assess whether certain retention functions are more likely with different numbers of intervals, perhaps because of the stability (or lack thereof) of the data. The descriptive information about the number of retention intervals is shown in Table 4.

#### Study design

Studies of retention and forgetting use either a between-subjects design (with a different group of participants at each retention interval), or a within-subjects design (measuring memory in all people at all retention intervals). This is important to account for because practice effects can occur with within-subjects’ designs, although it does reduce some error variance (Greenwald, 1976). In our corpus, within participant designs were coded as 0 and between participant designs as 1. There were more within-participant designs (*n* = 489) than between (*n* = 294).

#### Number of observations

We also include the number of observations per participant. This includes the number of trials. The more observations there were per person, the more stable the data are likely to be. Thus, more observations are more likely to capture retention, and provide more replicable and stable results. The number of observations per person can compensate for low levels on other factors, such as the number of participants (Smith & Little, 2018).

#### Amount of data

We combined information about sample size and the number of observations per person to calculate the overall amount of data in each study. This is the number of participants in the study times the number of observations per person. Descriptive information about both the number of observations per person and the amount of data are provided in Table 4. However, for all further analyses, number of participants and number of observations per participant are not considered, as the amount of data measure was derived from these.

### Retention characteristics

#### Retention intervals

Memory exhibits different properties at different periods of time. Traditionally, there is a distinction between short-term/working memory and long-term memory (e.g., Atkinson & Shiffrin, 1968; Cowan, 2008). There have also been suggestions of other divisions, such as between long-term memory and long-lasting memory (McGaugh, 2000). These phases of memory may have different neurological and behavioral properties. For example, there is a shift in neurological processes over the span of retention, where the hippocampus is more active in early memory consolidation but becomes less active as memories are transferred to the neocortex (e.g., Squire & Alvarez, 1995). The neurological shift is reflected in changing speeds of forgetting (e.g., Radvansky et al., 2022), where forgetting speed decreases up to about a day, increases between 1 and 9 days, and remains somewhat stable thereafter.

Because there are shifts in the neural mechanisms supporting memory retention, it is possible that forgetting can be best described by different functions over the course of retention. As a hypothetical example, it could be that retention is better captured by a logarithmic function prior to one day, but a power function at longer delays. Thus, we include delay to allow us to discover any such differences. The issue of retention delay, while seemingly straight-forward, becomes somewhat thorny when considering the fit of data to retention and forgetting functions. There are many ways to quantify delay in studies with multiple delay intervals, so we capture delay in four ways: (1) the *shortest retention interval*, (2) the *longest retention interval,* (3) the *average retention interval,* and (4) the *retention range* (longest–shortest). For consistency, all studies are coded into the nearest reasonable number of seconds. Descriptive data on each of these is provided in Table 4.

#### Initial memory

Somewhat related to degree of learning is initial memory. Initial memory could affect the fit and rate of forgetting (Slamecka & McElree, 1983a, 1883b; Wixted, 2022). For example, Anderson and Schooler (1991) suggested that a logarithmic function has a steeper slope when there is a higher initial memory, but the power function does not depend on the initial memory. Thus, the best-fitting function may be related to initial memory levels. The descriptive data for initial memory levels are provided in Table 4.

Note that for some studies, the first memory assessment is reported as being made “immediately” or after 0 s. Rather than setting an initial delay time as 0 in these cases, which would literally mean that the information and the memory test were perfectly coexistent in time, and because some functions require a non-zero value to calculate, we took two approaches. The first was the acknowledgement that an immediate memory test was not actually instantaneous. Instead, there was some nominal delay, even if it is something like the refresh rate of a computer screen. To provide an estimate of what the actual delay time was we either used an estimate based on information about actual display durations or provided an educated estimate of how long instructions for a memory test would take (e.g., 30 s). We note in our corpus how these initial estimates were made in applicable cases. In some cases, a 0-second delay is more in line with what actually happened, such as in cases of short-term memory testing. This is a problem for some memory functions, such as the power function, because it is mathematically undefined at 0 seconds. To address this, we set the number of seconds for this initial memory at a small value (e.g., .01 s) that is not likely to be psychologically meaningful in the context of these data sets.

## Confirmatory and exploratory analyses

While our approach is largely atheoretical, there are some theoretical implications for what we find. Our analyses are part confirmatory and part exploratory. The confirmatory analyses address issues or findings that are already reported in the literature. In comparison, the exploratory analyses are those for which there is no strong, a priori, theoretical expectation or prediction about the outcome. However, these are of interest because they have the potential to provide insight to guide future research.

### Confirmatory analyses

#### Best-fitting functions

Of most interest to us here, we consider predictions for which of the retention and forgetting functions will best capture the data. Previously, Rubin and Wenzel (1996a, 1996b), in their extensive analysis, reported that logarithmic functions fit the data best most often, followed by power, exponential-power, and hyperbolic power, and with linear patterns doing the worst by far. This would be in line with some of the earliest work on memory retention and forgetting (i.e., Ebbinghaus, 1885). Moreover, they also suggest that this will be more likely to be the case for simpler materials, compared with complex memories, such as autobiographical memories.

A competing prediction, based on a report by Wixted and Ebbesen (1991a, 1991b), is that the data will be best fit by a power function. This is almost a default assumption of researchers studying retention and forgetting (e.g., Anderson & Schooler, 1991; Averell & Heathcote, 2011; Carpenter et al., 2008a, 2008b; Wickelgren, 1974; Wixted & Carpenter, 2007). An attraction of the power function is the idea that forgetting functions “almost invariably exhibit a decreasing relative rate of forgetting, as noted long ago by Jost (1897)” (Wixted, 2022, p. 1779). This invariability is assessed here.

#### Material characteristics

Rubin and Wenzel’s (1996a, 1996b) report predicts that autobiographical memories will be better fit by power functions than other types of materials. We can tentatively expand this to memory for any type of complex set of materials (e.g., stories). Rubin and Wenzel arrived at this conclusion largely based on a consideration of autobiographical memory studies using the Galton–Crovitz technique of eliciting memory reports (Crovitz & Quina-Holland, 1976). We exclude these here because this approach does not assess how *much* is remembered from a given time period, but only provide information about the memories initially retrieved in response to a cue.

A competing prediction is that autobiographical memories will be more likely to be well-fit by linear functions (Linton, 1982a, 1982b). This is based on the pattern of data observed for long-term memory for an extensive autobiographical memory study. A more general prediction is that as material complexity increases, the pattern of forgetting will be more linear (Fisher & Radvansky, 2019). This is based on research that has found clear and stable evidence of linear forgetting. Fisher and Radvansky’s (2019) report highlighted the fact that when a linear pattern of retention and forgetting is observed, the studies involved used more complex materials (e.g., narratives).

#### Learning characteristics

Another observed methodological factor involved in the pattern of retention and forgetting is the degree of learning. One prediction is that when there is a higher degree of learning, the pattern of forgetting will most likely be linear (Fisher & Radvansky, 2019). A review of the literature showed that when clear linear patterns of forgetting are observed, it is not unusual for such studies to involve a higher degree of learning, as with overlearning (e.g., Burtt & Dobell, 1925a, 1925b).

Having said all of this, we explicitly note here that we are aware that there may be other reports or accounts in the literature that provide a basis for confirmatory predictions that we have missed.

### Exploratory analyses

#### Year of publication

There are no strong a priori expectations that the year a study was published will influence the pattern of data. However, we do include this as a factor in our analysis to address the possibility that something, perhaps methodologically, has changed over the years to yield different patterns of memory retention and forgetting.

#### Sample size

There is no question that sample sizes can influence the patterns of data observed in psychological studies. Studies with small sample sizes may provide distorted views of the mind. Thus, it is reasonable to expect that differences in sample size may lead to different patterns of results, some of which may be more distorted than others. For example, if some memory functions are only seen with smaller sample sizes, then this would be an indication that that function is a result of more random fluctuations in the observed pattern of data, and not reflective of underlying memory mechanisms. Our analyses allow for this assessment.

#### Memory test characteristics

While there has been some suggestion that memory test types may influence the pattern of observed retention and forgetting (e.g., Haist et al., 1992), there may be aspects of the memory testing process itself that influence this pattern of which we are not aware. These aspects may include study design (such as whether it is a within- or between-participants design) and the number of observations per person.

#### Retention characteristics

The influence of various aspects of retention, such as the number of retention intervals, the shortest and longest intervals, the average interval, and the range of the retention interval, are largely unknown. Our analysis will provide some insight into this.

## Analyses

We have two basic analyses. The first is in line with the approach taken by Rubin and Wenzel (1996a, 1996b). Specifically, we compare the fits of the top four functions from Rubin and Wenzel’s work (logarithmic, power, hyperbolic-power, and exponential-power), along with the linear function in cases in which there is no net change over time or which memory is increasing, to assess how often each of these fit the data better than the others. Our second major set of analyses was to assess which of a wide range of factors are more likely to produce one pattern of data over another. That is, what aspects of the sample, the materials, the memory assessment, and so on, lead to different patterns of retention and forgetting.

### Best-fitting functions analysis

We first consider the assessment of how often each of our functions was the best fit for each of the data sets. As a reminder, Rubin and Wenzel (1996a, 1996b) fit all the data sets in their corpus to 105 functions as part of their primary analysis. They then tallied how often a given function was among the best 10 fitting functions for a given data set. From this, they concluded that the best-fitting four functions were logarithmic, power, exponential-power, and hyperbolic-power. We take a similar approach here, with some changes. Again, we deviate from Rubin and Wenzel in that we are not comparing 105 functions, as they did (they rejected many of them). We also deviated from Rubin and Wenzel in that while they only considered data when *r*^2^ ≥ .90 for at least one function, we allow for poorer fits. Second, while they treated each data set equivalently, we adjusted for the number of observations in a data set by using weighted means. It is important to account for the size of the study, in terms of the number of participants and the number of observations. Otherwise, studies with few participants/observations can place undue weight on the results and skew our conclusions. For those readers interested in the patterns of results when data sets were limited to *r*^*2*^ ≥ .90 and/or the data were not adjusted for the number of observations, these are available in our online Supplement C.

**Fig. 1:**
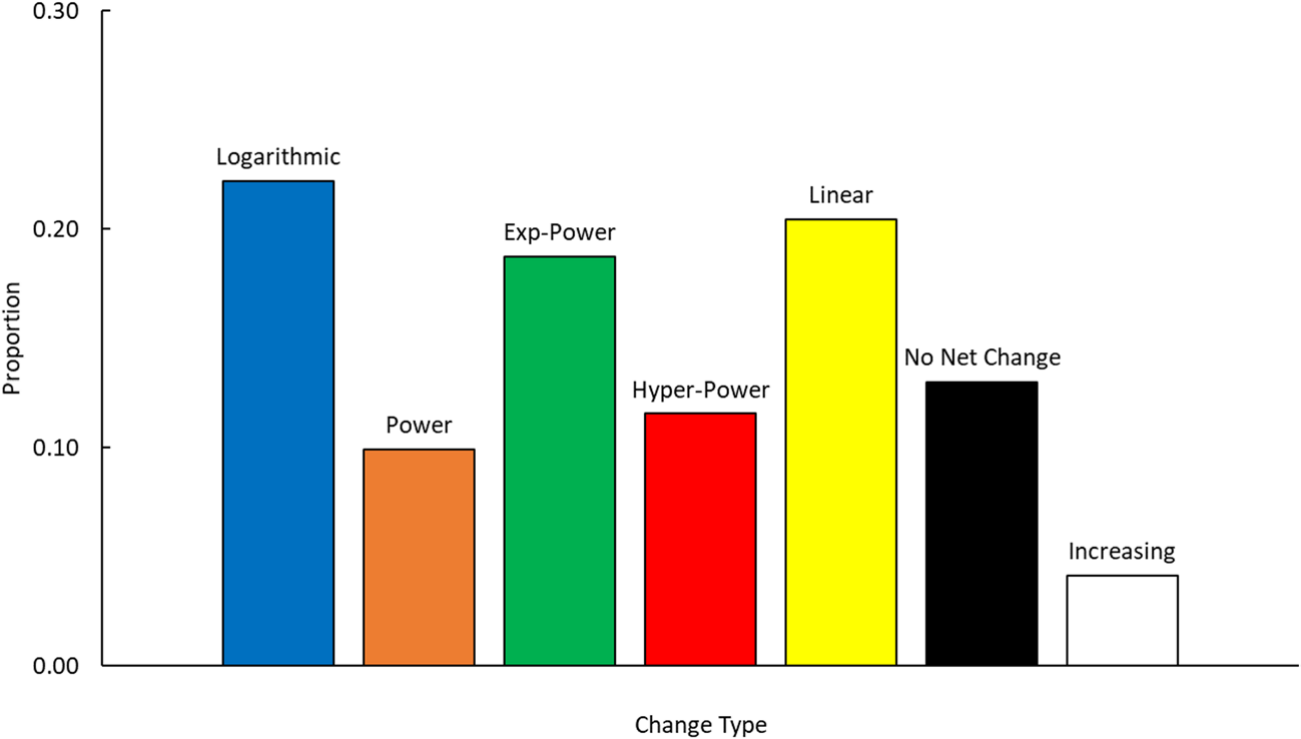
The distribution of categories along with an adjustment for the number of observations

The results of this analysis are in Fig. 1. As can be seen, the logarithmic function was the most successful because it best fit the largest proportion of data sets, followed closely by the linear and exponential-power functions. The hyperbolic-power function did worse, and the power function again did the worst. Finally, the relative proportion of studies that conformed to either no net change or increasing was substantial.

Overall, this analysis failed to support two confirmatory predictions. The first, based on work by Rubin and Wenzel (1996a, 1996b), was that logarithmic functions would do best, followed by power, exponential-power, and hyperbolic power, and with linear patterns doing the worst. Instead, we observed very different patterns of effectiveness of the various functions at capturing memory retention and forgetting.

The second unsupported prediction was that, based on a report by Wixted and Ebbesen (1991a, 1991b), as well as others, the dominant equation would be a power function. They might have observed those results for no other reason than because the averaging of various other underlying functions can be well fit by a power function (e.g., Anderson, 2001), particularly if there is greater variability among the individual forgetting functions due to variability in participants and memory for different material items. Based on this alone, one might expect power functions to do very well, even if the underlying pattern of memory loss is not a power function. However, with our corpus, the power function was the best to the smallest degree. Thus, prior implications of the dominance of the power function as the best description of memory retention and forgetting was not supported. At a minimum, given that power functions can be observed from an averaging of other functions with higher levels of variability in their loss rates, within studies, there is relatively little variability across participants and items.^5^

One other point to note is that goodness of fit values for the loss functions are correlated with one another, suggesting that some of them produce patterns that are difficult to distinguish with some memory data. The correlation matrix is shown in Table 6. As can be seen, the power and logarithmic function fits were highly correlated, as were the exponential-power and hyperbolic-power functions. The linear function differed the most from the other functions. Thus, there seem to be three families of functions here: (1) logarithmic/power, (2) exponential-power/hyperbolic-power, and (3) linear. One could drop consideration of the power and hyperbolic-power functions and still provide a fairly accurate characterization of most forgetting curves.

**Table 6:** Correlation of the fits for the five functions

	Logarithmic	Power	Exponential-Power	Hyperbolic-Power
Power	.98			
Exponential-Power	.82	.80		
Hyperbolic-Power	.82	.82	.99	
Linear	.55	.52	.87	.82

After assessing which functions fit the data most often, we also assessed how well the various functions did when they were the best fit for a data set. First, the exponential-power (*M* = .968, *SE* = .004; *min* = .764, *max* = 1.00) and hyperbolic-power functions (*M* = .966, *SE* = .006; *min* = .522, *max* = 1.00) fits did the best. This was followed by the power (*M* = .934, *SE* = .008; *min* = .651, *max* = 1.00), linear (*M* = .888, *SE* = .010; *min* = .510, *max* = 1.00), and logarithmic functions (*M* = .883, *SE* = .010; *min* = .514, *max* = 1.00).

#### Typical functions

**Fig. 2:**
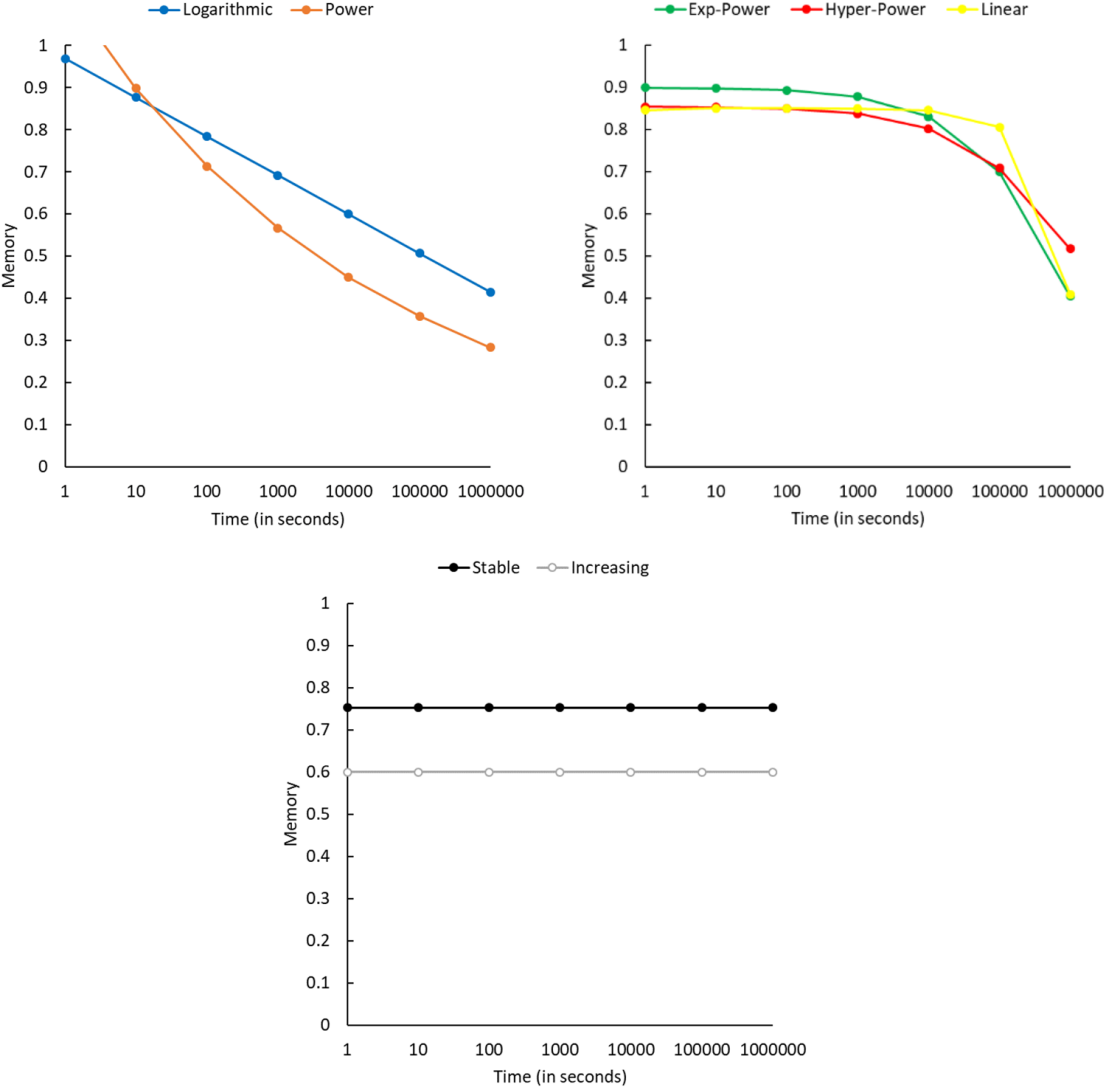
Typical pattern of data across log time for each of our categories. *Note.* For ease of understanding: 1 minute = 60 seconds, 1 hour = 3,600 seconds, 1 day = 8,640 seconds, 1 week = 604,800 seconds, 1 month (30 days) = 2,592,000 seconds, 1 year = 31,536,000 seconds. (Color figure online)

To get a feel for the different categories of memory retention and forgetting for our five functions, as well as no net change and increasing data sets, we plotted each of these using the median value for the *a* and *b* formula values. For the no net change data sets, we used the median initial performance level, and then extended that throughout. For the increasing data sets, we used the median *a* and* b* values for the best-fitting linear functions. The plots for these typical patterns are shown in Fig. 2. As can be seen, the logarithmic and power functions both show more dramatic loss over log time, especially for earlier retention intervals, with the power function showing more rapid loss. Also, the exponential-power and hyperbolic-power functions closely resembled each other, and the linear pattern was closer to these as well. Finally, the increasing data sets started out at a lower initial level of performance, overall, and there was very little change over time for the typical data set. Thus, many of these data sets could be grouped with the no net change data sets, without much loss in the accuracy of the characterization of the data.

## Memory factors analysis

Our next aim was to determine which factors contribute to different patterns of retention and forgetting. The first step was to assess whether any variables were strongly correlated with one another, and drop those that were to reduce redundancy. The next step was to assess the characteristics of the data sets for our seven patterns of performance over time. This may provide some insight into which study characteristics, when present, are likely to lead to a particular retention and forgetting pattern.

### Correlation analysis

Our first step was to assess whether any of our 13 numeric variables are strongly correlated with one another. The results of a correlation matrix are shown in Table 7. First, the correlation between Longest Retention Interval and Range was nearly perfect (*r* = .99). We elected to drop Range because, conceptually, we are interested in how long people remember things.

**Table 7:** Correlations among the various independent variables

	Year	Complexity	Multiple study?	Degree of learning	Distractor	Design	Amount of data	Num. of RI	Shortest RI	Longest RI	Average RI	RI range
Complexity	0.20											
Multiple study?	−0.20	0.17										
Degree of learning	0.11	0.77	0.52									
Distractor	−0.04	−0.18	−0.11	−0.12								
Design	−0.09	−0.01	0.08	0.06	0.07							
Amount of data	0.07	−0.04	0.02	0.03	−0.09	−0.26						
Num. of RI	−0.12	0.09	0.04	0.07	−0.09	−0.03	0.55					
Shortest RI	−0.01	0.24	0.37	0.27	−0.17	0.25	−0.09	0.04				
Longest RI	−0.05	0.27	0.46	0.31	−0.22	0.18	0.05	0.18	0.79			
Average RI	−0.04	0.28	0.45	0.32	−0.21	0.23	−0.02	0.14	0.88	0.98		
RI range	−0.05	0.26	0.45	0.30	−0.22	0.16	0.07	0.20	0.72	0.99	0.95	
Initial memory	−0.05	−0.12	0.14	0.00	0.01	−0.03	0.04	0.09	0.00	0.06	0.04	0.07

Moreover, we elected to identify variables that are correlated .70 or greater as collinear. To reduce collinearity, we also reduced the number of variables that met this standard criterion. Not surprisingly, the remaining three variables related to the length of the retention interval (Shortest, Longest, and Average Retention Interval), were highly inter-correlated (*r* = .72 to .98) and collinear. We elected to keep Longest Retention Interval. Another indicator of collinearity was between Complexity and Degree of Learning (*r* = .77). To address this, we elected to drop the Degree of Learning because it was also highly correlated with Multiple Study Opportunities (*r* = .52).

Finally, there was still one large correlation, between Amount of Data with Number of Retention intervals (*r* = .55). This is sensible. In general, the more retention intervals that were tested, the more data there was. This is largely unavoidable, and we retain these variables for our analyses but with an awareness of this relationship. Overall, we reduced the number of independent variables from 13 to 9.

#### Curve category characteristics

Our next step was to assess how our study characteristics differed across our seven categories. We did this in two ways. We first tested for any differences for each of the individual factors using ANOVA and Tukey tests. We then did logistic regressions in which we assessed the ability of our factors to predict when a given function would be the best at capturing the data sets.^6^ The factor means and standard errors for each of the memory patterns is shown in Table 8. Here, we do not report data from studies using anagram solution, matching, problem solving, and source monitoring measures because there were so few of them. Moreover, we collapsed stem and fragment completion studies because they were methodologically and conceptually so similar.

**Table 8:** Characteristic means of the data subsets (standard error in parentheses)

	Logarithmic	Power	Exp-Power	Hyp-Power	Linear	No net change	Increasing
Year	1992 (1.8)	1988 (2.7)	1993 (2.5)	1994 (2.1)	1998 (1.4)	1999 (2.5)	1990 (1.8)
Complexity	3.8 (0.2)	2.9 (0.2)	3.5 (0.2)	3.6 (0.2)	4.5 (0.2)	4.5 (0.3)	4.9 (0.2)
Multiple Study?	0.44 (.04)	0.27 (.04)	0.51 (.04)	0.40 (.04)	0.57 (.04)	.44 (.06)	.78 (.05)
Distractor	0.18 (.03)	0.20 (.04)	0.28 (.04)	0.17 (.03)	0.12 (.02)	.13 (.04)	.04 (.02)
Free Recall	.46 (.04)	.40 (.05)	.49 (.04)	.44 (.04)	.46 (.04)	.47 (.06)	.66 (.06)
Cued Recall	.13 (.03)	.14 (.03)	.19 (.03)	.16 (.03)	.11 (.02)	.09 (.04)	.12 (.04)
Recognition	.17 (.03)	.19 (.04)	.16 (.03)	.22 (.04)	.10 (.02)	.20 (.05)	.08 (.04)
Multiple Choice	.13 (.03)	.09 (.03)	.10 (.03)	.17 (.03)	.27 (.03)	.22 (.05)	.15 (.04)
Savings	.01 (.01)	.09 (.03)	.02 (.01)	.00 (.00)	.00 (.00)	.00 (.00)	.00 (.00)
Stem/Fragment Completion	.08 (.02)	.09 (.03)	.02 (.01)	.01 (.01)	.04 (.01)	.02 (.02)	.00 (.00)
Design	0.58 (.04)	0.73 (.04)	0.61 (.04)	0.73 (.04)	0.70 (.04)	.66 (.06)	.68 (.05)
Amount of data	3,838 data points (455)	2,584 (406)	4,504 (834)	2,732 (470)	3,638 (846)	6,413 (2765)	1,726 (413)
Num. of RI	5.0 (.20)	4.8 (.22)	4.4 (.16)	3.9 (.12)	4.7 (.17)	6.3 (1.06)	4.1 (.12)
Longest RI	1.900^8^ (3.327^7^)	4.581^7^ (2.150^7^)	9.707^7^ (3.113^7^)	4.694^7^ (2.115^7^)	4.795^8^ (5.142^7^)	4.916^8^ (8.254^7^)	6.933^8^ (7.206^7^)
Initial memory	.72 (.02)	.72 (.02)	.81 (.02)	.77 (.02)	.76 (.02)	.67 (.03)	.57 (.03)

ANOVA degrees of freedom are all 6, 856. *Complexity* uses our 7-level categorization described earlier, with 1 being least complex, and 7 being most complex. *Multiple study* was coded as either 0 (one exposure) or 1 (multiple exposures). For ease of understanding 1 minute = 60 seconds, 1 hour = 3,600 seconds, 1 day = 8,640 seconds, 1 week = 604,800 seconds, 1 month (30 days) = 2,592,000 seconds, 1 year = 31,536,000 seconds.

For the data in Table 8, we performed an ANOVA comparing performance on each of the factors (Table 9). If the ANOVA was significant (at least marginally), we also report any significant pairwise Tukey comparisons. One concern may be that five of these patterns are loss functions, and the other two (No net change and Increasing) are not. It might be that there are qualitative differences that result in memory loss versus not. Thus, we did each of our analyses twice, first including the No net Loss and Increasing pattern data sets, and then without. We report the first here, and the results are shown in Table 9. The second are available in our on-line Supplement D for interested readers. There were no major differences between the two analysis approaches.

**Table 9:** Individual characteristics results of overall ANOVA and Tukey test comparisons (comparisons that did not reach significance are not shown)

Factor	ANOVA	Tukey test results
Year	*F* = 2.82, *p* = .01, η_p_^2^ = .02	Power < Linear (*t* = 3.45, *p* = .01, *d* = .46) Power < No net change (*t* = 2.83, *p* = .07, *d* = .43)
Complexity	*F* = 10.53, *p* < .001, η_p_^2^ = .07	Power < Logarithmic (*t* = 3.28, *p* = .02, *d* = .39) Power < Linear (*t* = 5.86,* p* < .001, *d* = .72) Power < No net change (*t* = 4.86, *p* < .001,* d* = .77) Power < Increasing (*t* = 9.19, *p* < .001, *d* = .33) Linear > Logarithmic (*t* = 3.05, *p* = .04, *d* = .32) Linear > Exponential-power (*t* = 3.64, *p* = .005, d = .43) Linear > Hyperbolic-power (*t* = 3.34, *p* = .02, *d* = .38) No net change > Exponential-power (*t* = 3.06, *p* = .04, *d* = .48) No net change > Hyperbolic-power (*t* = 2.82, *p* = .07, *d* = .42) Increasing > Logarithmic (*t* = 3.89,* p* = .002,* d* = .54) Increasing > Exponential-power (*t* = 4.36, *p* < .001, *d =* .68) Increasing > Hyperbolic-power (*t* = 4.12,* p* < .001, *d* = .60)
Multiple Study?	*F* = 9.86, *p* < .001, η_p_^2^ = .07	Power < Exponential-power (*t* = 3.74, *p* = .004, *d* = .50) Power < Linear (*t* = 4.94, *p* < .001, *d* = .62) Power < Increasing (*t* = 1.16, *p* < .001,* d* = 1.17) Power < Logarithmic (*t* = 2.89,* p* = .06, *d* = .36) Linear < Increasing (*t* = 3.14* p* = .03,* d* = .44) Linear > Hyperbolic-power (*t* = 2.99,* p* = .04,* d* = .34) Increasing > Logarithmic (*t* = 5.04, *p* < .001, *d* = .70) Increasing > Exponential-power (*t* = 3.84, *p* = .003, *d* = .57) Increasing > Hyperbolic-power (*t* = 5.41, *p* < .001,* d* = .81) Increasing > No net change (*t* = 4.12, *p* < .001, *d* = .74)
Distractor	*F* = 4.34, *p* < .001, η_p_^2^ = .03	Increasing < Exponential-power (*t* = 4.47,* p* < .001, *d* = .63) Increasing < Power (*t* = 2.93,* p* = .05, *d* = .44) Increasing < Logarithmic (*t* = 2.73,* p* = .09, *d* = .37) Exponential-power > Linear (*t* = 3.78, *p* = .003, *d* = .42) Exponential-power > No net change (*t* = 2.71, *p* = .09,* d* = .37)
Free recall	*F* = 2.29, *p* =.03, η_p_^2^ = .02	Increasing > Power (*t* = 3.39, *p* = .01,* d* = .51) Increasing > Logarithmic (*t* = 2.93, *p* = .05,* d* = .40) Increasing > Linear (*t* = 2.94, *p* = .05,* d* = .41)
Cued recall	*F* < 1	None
Recognition	*F* = 2.25, *p* =.04, η_p_^2^ = .02	Hyperbolic-power > Linear (*t* = 2.73, *p* = .08, *d* = .32) Hyperbolic-power > Increasing (*t* = 2.73, *p* = .09, *d* = .39)
Multiple Choice	*F* = 4.56, *p* < .001, η_p_^2^ = .03	Linear > Power (*t* = 4.10, *p* < .001, *d* = .51) Linear > Exponential-power (*t* = 4.06, *p* = .001, *d* = .47) Linear > Logarithmic (*t* = 3.73, *p* = .004, *d* = .40)
Savings	*F* = 6.99, *p* < .001, η_p_^2^ = .05	Power > Hyperbolic-power (*t* = 5.36, *p* < .001, *d* = .70) Power > Linear (*t* = 5.63, *p* < .001, *d* = .70) Power > No net change (*t* = 4.40, *p* < .001, *d* = .70) Power > Increasing (*t* = 4.63, *p* < .001, *d* = .70) Power > Logarithmic (*t* = 4.95, *p* < .001, *d* = .61) Power > Exponential-power (*t* = 3.90, *p* = .002, *d* = .51)
Stem/Fragment Completion	*F* = 3.98, *p* < .001, η_p_^2^ = .03	Power > Hyperbolic-power (*t* = 3.19, *p* = .02, *d* = .42) Power > Increasing (*t* = 3.02, *p* = .04, *d* = .46) Power > Exponential-power (*t* = 2.85, *p* = .07, *d* = .38) Logarithmic > Hyperbolic-power (*t* = 3.24, *p* = .02, *d* = .37) Logarithmic > Increasing (*t* = 2.98, *p* = .05, *d* = .41) Logarithmic > Exponential-power (*t* = 2.84, *p* = .07, *d* = .33)
Design	*F* = 2.30, *p* = .03, η_p_^2^ = .02	Logarithmic < Hyperbolic-power (*t* = 2.87, *p* = .06, *d* = .33)
Amount of data	*F* = 2.03, *p* = .06, η_p_^2^ = .01	Increasing < No net change (*t* = 2.87, *p* = .06, *d* = .49)
Num. of RI	*F* = 5.30, *p* < .001, η_p_^2^ = .04	Logarithmic > Hyperbolic-power (*t* = 2.91,* p* = .06, *d* = .49) No net change > Power (*t* = 3.05, *p* = .04, *d* = .27) No net change > Exponential-power (*t* = 3.98, *p* = .001,* d* = .37) No net change > Hyperbolic-power (*t* = 5.07, *p* < .001, *d* = .46) No net change > Linear (*t* = 3.47, *p* = .01, *d* = .32) No net change > Increasing (*t* = 4.16, *p* < .001, *d* = .37) No net change > Logarithmic (*t* = 2.81, *p* = .07, *d* = .26)
Longest RI	*F* = 29.28, *p* < .001, η_p_^2^ = .17	Linear < Increasing (*t* = 3.21, *p* = .02, *d* = .32) Linear > Logarithmic (*t* = 5.62, *p* < .001, *d* = .51) Linear > Power (*t* = 7.21, *p* < .001,* d* = .78) Linear > Exponential-power (*t* = 6.80, *p* < .001,* d* = .68) Linear > Hyperbolic-power (*t* = 7.77, *p* < .001,* d* = .81) No net change > Logarithmic (*t* = 4.28,* p* < .001,* d* = .59) No net change > Power (*t* = 5.79, *p* < .001, *d* = 1.01) No net change > Exponential-power (*t* = 5.33, *p* < .001,* d* = .83) No net change > Hyperbolic-power (*t* = 6.05, *p* < .001, *d* = 1.05) Increasing > Logarithmic (*t* = 7.56, *p* < .001, *d* = .99) Increasing > Power (*t* = 8.85, *p* < .001, *d* = 1.47) Increasing > Exponential-power (*t* = 8.52, *p* < .001,* d* = 1.26) Increasing > Hyperbolic-power (*t* = 9.30, *p* < .001, *d* = 1.52)
Initial memory	*F* = 12.28, *p* < .001, η_p_^2^ = .08	Increasing < Logarithmic (*t* = 4.98,* p* < .001, *d* = .68) Increasing < Power (*t* = 4.51, *p* < .001, *d* = .68) Increasing < Exponential-power (*t* = 7.74, *p* < .001, *d* = 1.15) Increasing < Hyperbolic -power (*t* = 6.53, *p* < .001, *d* = .93) Increasing < Linear (*t* = 6.44, *p* < .001,* d* = .86) Increasing < No net change (*t* = 2.93, *p* = .06, *d* = .45) Exponential-power > Logarithmic (*t* = 3.80, *p* = .003, *d* = .45) Exponential-power > Power (*t* = 3.32,* p* = .02,* d* = .48) Exponential-power > Stable (*t* = 4.06, *p* = .001,* d* = .63)

The results of the regression analyses are shown in Table 10.

**Table 10:** Results of logistic regressions for each of the characteristics

	Logarithmic	Power	Exp-Power	Hyp-Power	Linear	No Net Change	Increasing
Year					*p* = .004; *z* = 2.87		
Complexity		*p* = .03; *z* = −2.13					
Multiple study?		*p* < .001;* z* = −3.61					
Distractor			*p* = .02; *z* = 2.41				
Free recall							
Cued recall							
Recognition							
Multiple choice							
Savings		*p* = .09; *z* = 1.70					
Completion							
Design	*p* = .01; *z* = −2.55			*p* = .01; *z* = 2.56			*p* = .02; *z* = −2.35
Amount of data			*p* = .08; *z* = 1.76				
Num. of RI	*p* = .03; *z* = 2.15			*p* = .01; *z* = −2.45		*p* = .03; *z* = 2.19	*p* = .003; *z* = −2.97
Longest RI		*p* = .09; *z* = −1.70	*p* = .002; *z* = −3.11	*p* < .001; *z* = −4.18	*p* < .001; *z* = 3.62	*p* < .001; *z* = 3.39	*p* < .001; *z* = 4.47
Initial memory			*p* < .001; *z* = 4.25		*p* = .06; *z* = 1.86	*p* = .01; *z* = −2.55	*p* < .001; *z* = −5.69

#### Logarithmic

According to the logistic regression, the logarithmic function was more likely to be the best fit for studies using a *between-participants design*. This was also only mildly observed with the individual characteristics analysis, in which the only significant difference was with hyperbolic-power functions, and that was marginal. Still, the fact that logarithmic functions were quite frequent, and many studies used between-participant designs, leads to the conclusion that this may be driving this high frequency.

The regression also revealed that logarithmic functions were likely to have *more retention intervals*. However, in the individual factor analyses, the only difference is with hyperbolic-power functions. Thus, it may be the case that logarithmic functions are more likely to emerge when researchers test a broader range of retention intervals, however the evidence is not strong.

Finally, it seems as though logarithmic functions are more likely when *savings* is used, but this is not seen with the individual characteristics analyses. Conversely, there is some evidence that logarithmic functions are more likely with *word stem/fragment completion tasks*, but this is only seen in the individual comparison analyses, and it is weak there.

#### Power

When power functions best fit a data set, the factor that was most strongly associated was *Multiple Study*, when people had been exposed to the materials once rather than multiple times, as seen in both the individual characteristic and regression analyses. Thus, power functions may be capturing weaker memories that a person has only experienced once.

This is supported by the finding that the data sets best fit by a power function also involved *less-complex materials*, which was also supported to some degree in the individual characteristics analysis. Thus, power functions are more likely to be seen with simplistic materials.

Moreover, like the logarithmic function, there was some weak evidence that power functions were likely to be the best-fitting functions when *savings* was the measure of memory, such as Ebbinghaus’s work. That said, this should be taken with a grain of salt given that there are so few studies in our corpus that used savings. Conversely, like logarithmic functions, there is some evidence that power functions are more likely with *word stem/fragment completion tasks*, but this is only seen in the individual comparison analyses, and it is weak there. Finally, there was some evidence that power functions were more likely to be the best when the data come from *older studies*, but, again, evidence for this is weak.

#### Exponential-power

A higher level of *initial memory* was by far the clearest factor to lead to exponential-power functions succeeding as the best. This was true both in the regression and individual characteristic comparisons. Thus, this sort of function is more likely to emerge with well-learned materials.

Furthermore, exponential-power functions were more likely to be the best when there was a *distractor task* just after learning. Again, there was support for this in both regression and individual characteristics comparisons, although weaker in the latter. A distractor task immediately after presentation of high initial memory likely disrupts consolidation processes.

In addition, exponential-power functions were the best-fitting ones when the *retention intervals* were relatively short, according to the regression. However, in the individual comparisons, exponential-power functions do not strikingly differ from the rest. This may be because there is so much overlap with other, especially curvilinear, functions.

Finally, there was some evidence that an exponential-power function was likely to be the best when there was *more data* in a data set, but this evidence was weak, and not evident in the individual characteristics analyses.

#### Hyperbolic-power

The factor that was most clearly associated with hyperbolic-power functions best-fitting a data set is *Longest RI*, when the retention intervals were relatively short, at least for the regression. There was a small amount of support for this in the individual characteristics analyses. Thus, these sorts of functions are more likely to be the best ones over shorter periods of time.

In addition, these functions were more likely to be the best when there were *fewer retention intervals*. This was evident in both the logistic regression and individual comparison results. It may be the case that this type of function, which is rarely discussed, may be more likely to be the best one when there are fewer data points to fit the curve, which lowers our confidence in it.

There was also some suggestion that this pattern was more likely to be the best when a *repeated-measures design* was used with the same people being tested at multiple time points. However, this was only significant in the regression. Finally, there was some suggestion that hyperbolic-power functions were more likely to be best fitting when *recognition* was used, but this finding was weak.

#### Linear

The factor that was most strongly associated with linear functions being the best fit for a data set was *Longest RI*, when the retention intervals were relatively long. This was the case both for regression and individual characteristic comparisons. Thus, those memories that are particularly long-lasting, such as autobiographical and event model memories, have a different memory retention and loss profile than other types of material. This finding also works against the idea that linear forgetting is a scaling artifact in which curvilinear forgetting would be observed if data across a longer time scale were collected (Wixted, 2022). If this were the case, then one would expect linear forgetting to be observed with shorter retention intervals, not longer.

This is supported to some degree by the finding that in the individual characteristic analyses, data sets that were best fit by linear functions tended to also involve *more complex materials*. That is, there was significant difference when compared against all the other loss functions. That said, this was not significant in the regression.

Another outcome was the effect of *year* in the regression, with the individual characteristic comparisons highlighting the difference between when linear and power functions were the best fits. This suggests that there is some aspect of those studies that is not well captured by other factors used here and which would need to be resolved by further research.

Also, there was some evidence that a linear function was likely to be the best-fitting one when there was greater *initial memory*, but only marginally so, but not for the individual characteristic comparisons. Thus, the evidence here is weak. Finally, there was a significant difference in the individual characteristic comparisons suggesting that linear forgetting patterns were more likely to be the best when *multiple choice* measures were used, although this was not significant in the regression, the reasons for this pattern not clear.

#### No net change

The factor that was most strongly associated with no net change across retention intervals, like linear patterns, was *Longest RI*, with relatively long retention intervals. This was the case in both the regression and individual comparison analyses. Thus, those memories that are longer-enduring are also less likely to show evidence of forgetting. This is akin to the idea of a memory permastore for long-lasting memories (e.g., Bahrick, 1984a, 1984b). Moreover, no net change was more likely to be observed in studies with *more retention intervals*, consistent with the idea that memories that are likely to be observed across multiple time periods are also likely to be more durable.

There was also some suggestion in the regression analysis that no net change was more likely to be the best solution when *initial memory* levels were relatively low, perhaps because most of forgetting processes had already occurred. This, however, was not supported by the individual characteristic comparisons. Finally, although not significant in the regression, the individual characteristic comparisons suggested that these patterns of data were more likely to involve *more complex memories*.

#### Increasing

The factor that was most strongly associated with increasing memory over time, like linear forgetting and no net change, was *Longest RI*, when the retention intervals were relatively long. This was found in both the regression and individual characteristic analyses. This is again consistent with the idea that this is more likely with materials that are durable in memory.

Unlike linear forgetting and no net change, data that were best described as increasing were more likely to have *initial memory* levels that were lower and included fewer retention intervals. This was found in both the regression and individual characteristic analyses. This makes sense in that these are likely to involve cases where there is more room for improvement. On top of this, this was more likely to happen, based on the regression, when there were *fewer retention intervals* and with *repeated-measures designs*, suggesting that some element of hypermnesia may be involved (e.g., Payne, 1987). That said, there was less support for this in the individual characteristics analyses.

Finally, there were several individual comparisons that were not significant in the regression. Specifically, increasing data patterns were more likely to involve *more complex materials*, *multiple study exposures*, the presence of a *distractor task* (from which recovery after initial encounter might be possible), and with *recall tests* (which is also where hypermnesia effects are more likely to occur).

#### Overview summary

At this point, let’s take a step back and consider why different factors may lead to different retention functions. All of these ideas are speculative, and would require explicit experimental investigation to support or refute them. Logarithmic and power functions are highly correlated. They are more likely to involve older studies with simpler materials and a single exposure. These sorts of forgetting functions may emerge because less complex memory traces can degrade more quickly, leading to clearly curvilinear forgetting functions that would be better captured by logarithmic and, especially, power functions. They were also more likely to be observed when the memory tasks involved savings or completion tasks, both of which place a heavier emphasis on implicit memory.

Much of this is reinforced by the regressions, especially for power functions, which were the best fits when there were single study opportunities, less complex materials, shorter retention intervals, and savings was used as the memory measure. In comparison, logarithmic functions were the best fit when between participants designs were used (which reduces the impact of learning the task, and the benefit of prior retrievals), and more retention intervals (perhaps because it would be more likely that an asymptote would be approached).

Exponential-power and hyperbolic-power functions were also similar, with exponential-power functions having clearer characteristics. Specifically, they were more likely to involve distractor tasks and higher levels of initial memory. The distractor tasks may disrupt consolidation processes, leading to more rapid forgetting earlier on. With the higher levels of initial memory, this may provide some resistance to forgetting processes earlier on, but which exert themselves after a period, leading to curvilinear patterns more like to those observed with data best fit by logarithmic and power functions.

The logistic analysis supported the idea that shorter retention intervals are more important for these functions. On top of this, exponential-power functions were also more likely to be the best fits when a distractor task was involved and higher initial levels of memory were recorded. They were also more likely with more data in a study and multiple study opportunities. Hyperbolic-power functions were more likely to be best-fitting functions when repeated-measures designs were used (allowing some influence of prior retrieval attempts), along with fewer retention intervals in the data set.

Linear functions were characterized by many of the same factors that also characterized stable and increasing data sets. These factors closely match those suggested in other work (Fisher & Radvansky, 2019). Specifically, it has been suggested that linear forgetting is more likely to be observed with complex materials that have been learned well. The additional finding that data sets with longer retention intervals fit along nicely with this given that more complex materials, such as memories for events, are likely to be more enduring. The additional characteristic that increasing data sets have lower initial memory is consistent with the idea that in order for memory to improve over time, the worse it is to begin with and the more room there is for improvement. Stable data sets might have more retention intervals on average because they are extended into longer periods of time, when memory traces are more likely to have been well-established.

In terms of the regressions, while complexity was not a significant predictor for any of these three patterns, all of them had longer retention intervals as a significant predictor. However, while linear forgetting was more likely to be observed with higher initial memory, stable and increasing data sets were more likely to have lower initial memory. Moreover, while stable memory data sets were more likely with more retention intervals, increasing memory data sets were more likely to involve fewer. Also, while stable data sets were more likely with single study opportunity data sets, increasing data sets were more likely to involve repeated-measures designs, suggesting that some element of hypermnesia may be involved. Finally, the finding that linear best-fitting functions were more likely to be observed for more recent studies suggest that there are aspects of these studies that are not well-captured by the factors that we identified here.

As a further step, we elected to create a guide for predicting the function of best fit for future work. As previously mentioned, one of our goals is to improve our ability to model and predict the amount of information retained in memory. This model allows researchers to input the characteristics of a study and obtain the expected function that would best predict memory over time for the study’s specific set of characteristics. Importantly, the results of the model should be approached with caution, as it is trained using datasets that are potentially underpowered, noisy, or biased. The model was created using the ChefBoost: C4.5 machine learning model, which works as a statistical classifier (Quinlan, 2014; Serengil & Teknoloji, 2021). The resulting model is available as Supplement E.

Overall, it is clear from our analyses that different functions describe changes in memory over time (or not, if memory is stable). These different functions tend to be associated with different material and task properties. Thus, as we continue to develop our understanding of when and why different functions are likely to be observed over time, we can better understand the mental representations and processes involved.

## General discussion

One of the most fundamental features we know about memory is that it changes over time (Ebbinghaus, 1885). If we hope to be able to predict future memory, we need to be able to provide an adequate description of the nature of that change over different types of information and over different time scales. Our aim was to identify the shape of the patterns of memory change over time, and to begin to identify characteristics that bring about different patterns of memory change.

There were a number of major findings to come out of our analyses. The first is, consistent with Rubin and Wenzel (1996a, 1996b), that there is no single function that captures the progress of memory retention and forgetting over time. Second, the functions varied noticeably in terms of the proportion of data accounted for best by a function, with logarithmic, exponential-power, and linear functions accounting for the bulk of the cases in which there was forgetting. This is consistent with the idea that different types of memory representations and processes can lead to different changes in memory over time (or even no change at all). Thus, such patterns can be used to further assess the nature of memory. Third, there was a strong similarity between logarithmic and power functions, as well as between exponential-power and hyperbolic functions. Linear functions differed from these. Next, let us consider some of the confirmatory and exploratory analyses discussed in the introduction.

The analysis of our corpus for retention and forgetting provides a means of addressing a wide range of confirmatory and exploratory analyses. If we know how various factors contribute to the goodness-of-fit for the various functions, it helps us predict memory from a subset of retention intervals. For example, if a linear function is best for factors *X*, *Y*, and *Z*, and a study has those factors, a linear function should be used to fit performance and predict future memory.

### Confirmatory analyses

#### Best-fitting functions

The first issue our analyses revealed is the relative prevalence of different function types in our corpus. Our assessment of the common function types is at odds with what has been reported in the literature. In the earlier meta-analysis by Rubin and Wenzel (1996a, 1996b), it was suggested that logarithmic functions would fit the data the best, followed by power, exponential-power, and hyperbolic-power, and with linear patterns doing the worst. However, we found that a different order emerged. While we are in line with Rubin and Wenzel in finding that logarithmic functions did the best, with exponential-power functions also doing well, we found that linear functions also accounted for a substantially larger proportion of the data. Moreover, power and hyperbolic-power functions, while accounting for some of the data sets, did more poorly relative to the first three.

Part of the reason for this may lie in how Rubin and Wenzel (1996a, 1996b) analyzed the data compared with how we did. Specifically, Rubin and Wenzel gave credit to a function if it was one of the top 10 (out of 105) best-fitting for a data set. In comparison, we are strictly looking at which was the best. Because logarithmic and power function fits are so highly correlated, when one of these does a good job, the other is likely to do so as well. Thus, they are both likely to make the top 10 with Rubin and Wenzel’s approach, but only one will be the actual best, as in our approach. The same can be said for exponential-power and hyperbolic-power functions.

Furthermore, Rubin and Wenzel (1996a, 1996b) did not consider whether data sets had data that either had no net change or were increasing, whereas we did. Such data sets may have been more likely to be excluded from their analysis because of their criterion of *r*^*2*^ ≥ .90 for at least one function. Such data sets, because they deviate from a traditional loss pattern, are less likely to fit one of these functions well. Thus, there is a substantial subset of data that are producing patterns over time that are not being well accounted for.

We now turn to other issues raised in the introduction. It has also been suggested that power functions best capture the pattern of forgetting (e.g., Wixted & Ebbesen, 1991a, 1991b). However, while power functions did the best for some data sets and were highly correlated with logarithmic functions, which did the best overall, they accounted for a smaller proportion of data sets compared with our other functions. This is particularly surprising given that there have been several studies showing that power functions should be quite common because averaging across other types of functions (as might occur when averaging across multiple memory traces) generally produces a power function as an artifact (e.g., R. B. Anderson, 2001). Moreover, the acceptance of a power function in the literature may be a consequence of unfortunate sampling error of a small set of studies from those assessments that have looked at multiple data sets (e.g., Wixted & Ebbesen, 1991a, 1991b).

Overall, our analyses suggest that there are clear regularities in how memory operates that in turn produces other types of common functions such as logarithmic, exponential-power, and linear functions. Logarithmic functions are intuitively sensible. Specifically, a simple logarithmic function conveys a constant proportion loss of information across a given unit of time. Similarly, a simple linear function conveys a constant amount of loss of information across a given unit of time.

The exponential-power function is less intuitive, and deserves further consideration. At the outset, it should be noted that the square root of time that is used in this function reflects a setting of the exponent of the power function of this formula to .5 (after Wickelgren, 1974). The more expanded version has an additional parameter: $M=a{e}^{-b*{t}^{-c}}$. Thus, this three-parameter function puts it outside of the set of two-parameter functions that were explored here and by Rubin and Wenzel (1996a, 1996b). That said, further research may show that such an approach better captures memory over time, despite the increase in the number of parameters. For the time being, like Rubin and Wenzel, we considered the most preferred two-factor functions.

#### Material characteristics

Our analysis helps clarify why, in some cases, forgetting seems to follow one pattern over another. That is there are some factors that influence the observed patterns of change. Rubin and Wenzel (1996a, 1996b) suggested that autobiographical memories will be more likely to be well fit by power functions, and we expanded this hypothesis to memory for complex materials more generally. Our analyses failed to support this prediction. This discrepancy may result from the fact that Rubin and Wenzel considered studies that largely used the Galton–Crovitz technique in which people respond with the first autobiographical memory they retrieve in response to a cue word (e.g., cake). These first retrieved memories may exhibit different properties than assessments of accuracy over various retention intervals.

In contrast to this, our analyses found that complex materials (Fisher & Radvansky, 2019), including autobiographical memory studies (e.g., Linton, 1982a, 1982b) are more likely to be better fit by a linear function. We were able to provide some confirmatory support for this finding.

#### Learning characteristics

Our analyses also partially supported the idea that when there are higher degrees of learning there are more linear patterns of forgetting (Fisher & Radvansky, 2019). This finding was present, and provided some confirmatory support, albeit this was weak.

### Exploratory analyses

#### Year of publication

Although we did not expect to find any influence of the year of publication on the patterns of forgetting, there were some significant effects. Specifically, it was found that older publications were more likely to conform to power functions than more recent work, which were more likely to conform to linear functions. This may be because older studies tended to emphasize simpler materials and newer studies more complex materials. This may contribute to differences in findings between our study and the one by Rubin and Wenzel (1996a, 1996b).

#### Amount of data

We saw very small influences of the amount of data on the observed pattern of retention and forgetting. Thus, there does not appear to be any strong evidence for the idea that some patterns of data may be observed because of fewer data points. This may be the case with hyperbolic-power functions. There was also some evidence that studies with more data were more likely to either be best fit by an exponential-power function or have no net change. However, this evidence was weak, and we would caution against using this as a basis of prediction.

#### Memory test characteristics

In our data set, there was some suggestion of an association of free recall tests with increasing functions and recognition with hyperbolic-power functions, although this evidence is very weak. There was also some suggestion that studies that used multiple choice tests were more likely to be associated with linear functions, and a strong suggestion that studies that used savings were more likely to be associated with power functions. Thus, there is some evidence to suggest that different memory tests may lead to different patterns of results. However, this was really only observed in the individual characteristic comparisons, and not when other factors were considered, as with the regression analyses.

#### Retention characteristics

Finally, our exploratory analysis of retention characteristics assessed the number and length of the retention intervals. In terms of the number of retention intervals, more retention intervals were associated with logarithmic and no net change patterns. In the first case, this may be because more retention intervals make it more likely that an asymptote would have been approached, making it difficult to detect any kind of changes over those time intervals. In comparison, data sets with fewer intervals were associated with hyperbolic-power and increasing power functions. The first, our most unusual function, may be reflective of studies with fewer time points, allowing for an unusual function to fit best by chance. The second is likely to emerge under more constrained circumstances, making it more likely for random variation to emerge as an increase in memory over time.

In terms of the longest retention interval, this is probably the one factor that was the clearest indicator of which pattern would be observed. Specifically, shorter retention intervals were better fit by curvilinear functions, such as logarithmic, power, exponential-power, and hyperbolic-power functions, whereas longer retention intervals were more likely to be better fit by linear functions, as well as being more likely to involve either no net change or be an increasing data set. Why might this be the case? It may be that, consistent with work by Ebbinghaus (1885), the largest changes in memory are more likely to occur early on after learning. Thus, when data sets are more likely to involve short retention intervals, these large changes are more likely to be observed. However, when studies focus on longer delays, this would not be revealed, with the data falling in a more consistent way, or even perhaps having reached an asymptote. If the information from these studies were sampled at shorter time intervals, more curvilinear patterns would be observed.

However, there are some important things to note. The first is that if a function holds true across time, it should not matter where along the curve it is sampled. So, a change in best-fitting pattern for different time periods is important. It suggests that memory processes are changing over time. Second, it is possible to observe patterns of data shifting from curvilinear to linear within the same time frame. For example, in a study by Fisher and Radvansky (2022a, 2022b), people memorized lists of sentences that varied in terms of the degree of learning. Although the same memory test delays were used in all conditions, the less the degree of learning, the better the data were fit by a power function, and the greater the degree of learning, the better the data were fit by a linear function. The same can be said for studies of the testing effect (e.g., Roediger & Karpicke, 2006a, 2006b). Thus, while length of retention interval was an important factor in our analyses of our corpus, it is not definitive.

### Unresolved issues

Our analysis here covered a wide range of issues. However, there are still many left unresolved. One of these is that recent work has suggested that memory retention and forgetting is not continuous, but goes through different phases (Radvansky et al., 2022). It might be the case that different functions are more appropriate for different time intervals after learning. Another is that different types of materials and methods may lead to differences in the rate with which information is forgotten (or not). This is a long-standing issue (Loftus, 1985a, 1985b; Rivera-Lares et al., 2022; Slamecka, 1985; Slamecka & McElree, 1983a, 1983b; Wixted, 2022) that data sets such as ours may be able to provide important insight into. We are actively pursuing both issues with our data set. It is also almost certainly the case that there may be aspects of memoranda and methods that influence patterns of retention and forgetting that have not been considered here.

## Conclusions

This work provided an opportunity to expand on the work originally reported by Rubin and Wenzel (1996a, 1996b). Our attempt uses a wider range of studies that is, in some ways, more inclusive, allowing for a broader assessment of memory retention and forgetting. We explored the degree to which different functions fit the available data, and the factors that contribute to those functions. This approach allows use to perform a wide range of confirmatory and exploratory analyses. This work has the potential to have a broad-ranging impact on psychological science.

Perhaps the most important issue addressed here is changes in memory over time. As is emphatically clear, like Rubin and Wenzel (1996a, 1996b), we did not find any evidence for a single function that does a good job with all of the data. Instead, we found the data when there was forgetting were best captured by (a) logarithmic/power functions, (b) exponential-power/hyperbolic-power functions, and (c) linear functions. Moreover, a sizable minority of studies in our corpus showed either no evidence of a net change across all of the retention intervals, or actually showed more than trivial improvement in performance over time. These different patterns of performance are almost certainly due to different underlying memory processes. Just what these are is a task left to future research.

Having identified which pattern best captured each of our data sets, we then assessed what study characteristics were more or less likely to lead to different patterns of performance. While we found that there were many characteristics associated with each pattern, they varied in the degree to which they served to distinguish one from another. Perhaps the strongest characteristic was the duration of the retention intervals, with shorter intervals being more likely to produce a curvilinear loss function, and longer intervals being more likely to produce linear, no net change, and improvement patterns. Our hope is that future work can use this as an inspiration to explore various theoretical ideas about the different patterns that cognitive and neurological processes are likely to produce and why they would do so.

## Supplementary Information

Below is the link to the electronic supplementary material.

Supplementary file1 (PDF 117 KB)

Supplementary file2 (PDF 143 KB)

Supplementary file3 (PDF 304 KB)

## Data Availability

All data used for our analyses are availble on-line at https://osf.io/wq9ty.

## Footnotes

1 Like Rubin and Wenzel (1996a, 1996b), we only consider two-parameter functions. Wickelgren (1974) also proposed a three-parameter function that is a variation of the power function. In addition to the *a* and *b* parameters, there is a third parameter, *c*, that is set at the level of memory performance at time 0. The equation is *M* = *c*(*at* + 1)^-*b*^ . With an extra parameter, this formula better fits some data sets than any two-parameter function. We do not consider it here for several reasons. First, this requires additional information, namely memory at time 0, which is simply not available for many of our data sets. Second, in some cases, memory at time 0 can be set to 1, but not in all cases. Finally, in some cases where an estimate of memory is provided at time 0, we can address this using a very small value for time close to, but not equaling 0, as with the power function.

2 As noted by Rubin and Wenzel (1996a, 1996b), the retention function that Ebbinghaus proposed, *M* = 100 - *a*/{[log(*t*)]^*b*^ + *a*}, is actually a combination of logarithmic, power, and hyperbolic components.

3 Signal detection measures of memory are not included because they do not capture the amount of information forgotten over time. As noted by Rubin and Wenzel (1996a, 1996b), signal detection measures of memory follow a different pattern of forgetting, which would introduce a confound.

4 Many studies reported data in a figure. To extract the data, we used Web Plot Digitizer (Rohatgi, 2019).

5 Note that for the studies with poor fits that were removed from our analyses, the power function was the best-fit one most often.

6 Memory test types (e.g., free recall, cued recall) were dummy coded for these analyses.
