# Supplementary material for: Memory from nonsense syllables to novels: A survey of retention
Source: Psychon Bull Rev. 2024 May 7;31(6):2437–64. doi: 10.3758/s13423-024-02514-3 (PMC11680664; doi:10.3758/s13423-024-02514-3)
Supplement: Supplementary file 1 — Supplementary file1 (PDF 117 KB) [file 13423_2024_2514_MOESM1_ESM.pdf]

Supplement C – Other Analyses of Best Fitting Function Distributions

In addition to the analysis reported in the paper, in which data were included even if the best fitting r^2 was less than .90 and there was an adjustment for the amount of data, here are three other distribution analyses for the various best fitting function categories. These include (a) when the analysis is restricted to r^2 = .90 or better for the best fitting function and there is no adjustment for the amount of data, (b) when there is no restriction to r^2 = .90 or better for the best fitting function and there is no adjustment for the amount of data, (c) when there was a restriction to r^2 = .90 or better for the best fitting function and there is was an adjustment for the amount of data.

Assessment 1 (.90 or better, no adjustment). This analysis was most like Rubin and Wenzel's (1996) by including the r^2 = .90 or better criterion, or falling into the no net change or increasing categories, and treating each data set as equal (*n* = 682). We deviated from Rubin and Wenzel's approach in that we also consider data sets with three or four retention intervals, and not just five or more. Moreover, we are not looking for whether a function was one of the top ten best out of 105, but simply whether it was the best. As can be seen in Figure 1, the exponential-power and hyperbolic-power functions fit the largest proportion of data sets, closely followed by the logarithmic and linear functions, with the power function accounting for a smaller proportion of studies. It should also be noted that there were a substantial portion of data sets (20%) that exhibited evidence of either no net change over time, or increasing memory over time.

Figure S1

The distribution of categories when only fits of .90, or were stable or improving data sets, are considered and there is no adjustment for the number of observations in each data set.

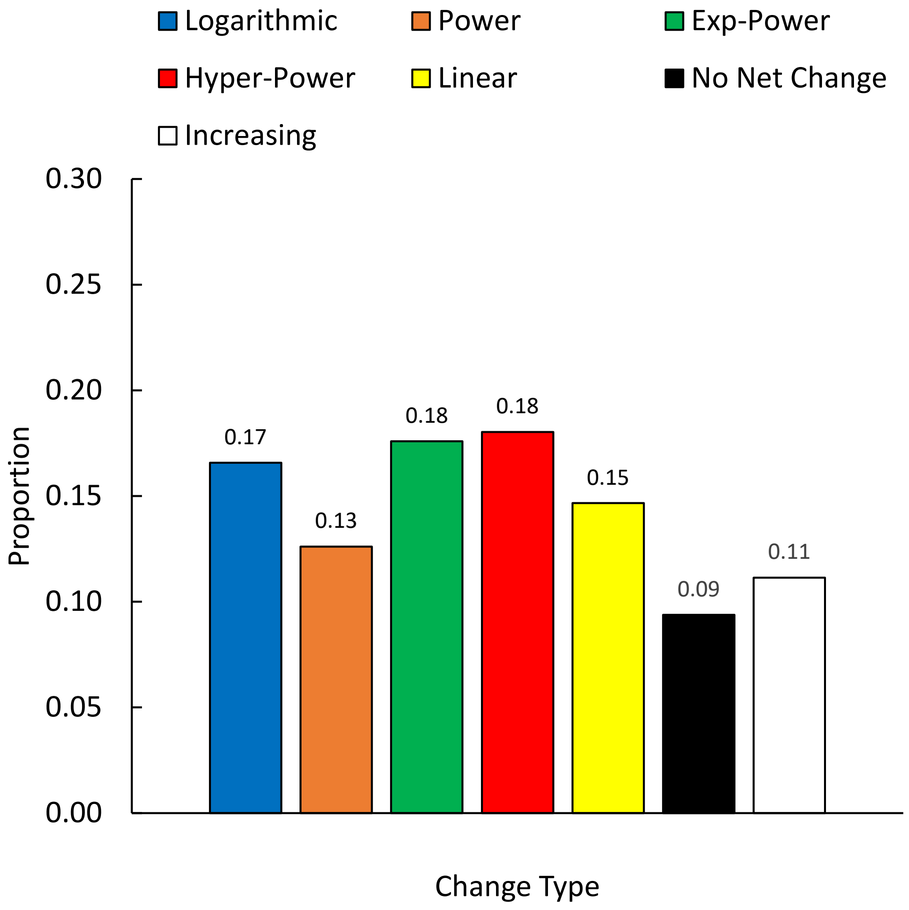

Assessment 2 (all data, no adjustment). This is a modification of the prior analysis in which we removed the criteria of r^2 = .90 or better (n = 873). As can be seen in Figure 2, the Logarithmic function was now the best, followed closely by the linear function. The exponential-power and hyperbolic-power functions did less well, and the power function again accounted for the smallest proportion of studies. The relative proportion of studies account for by no net change and increasing data sets was reduced (16%), as would be expected.

Figure S2
The distribution of categories when all data sets are considered and there is no adjustment for the number of observations.

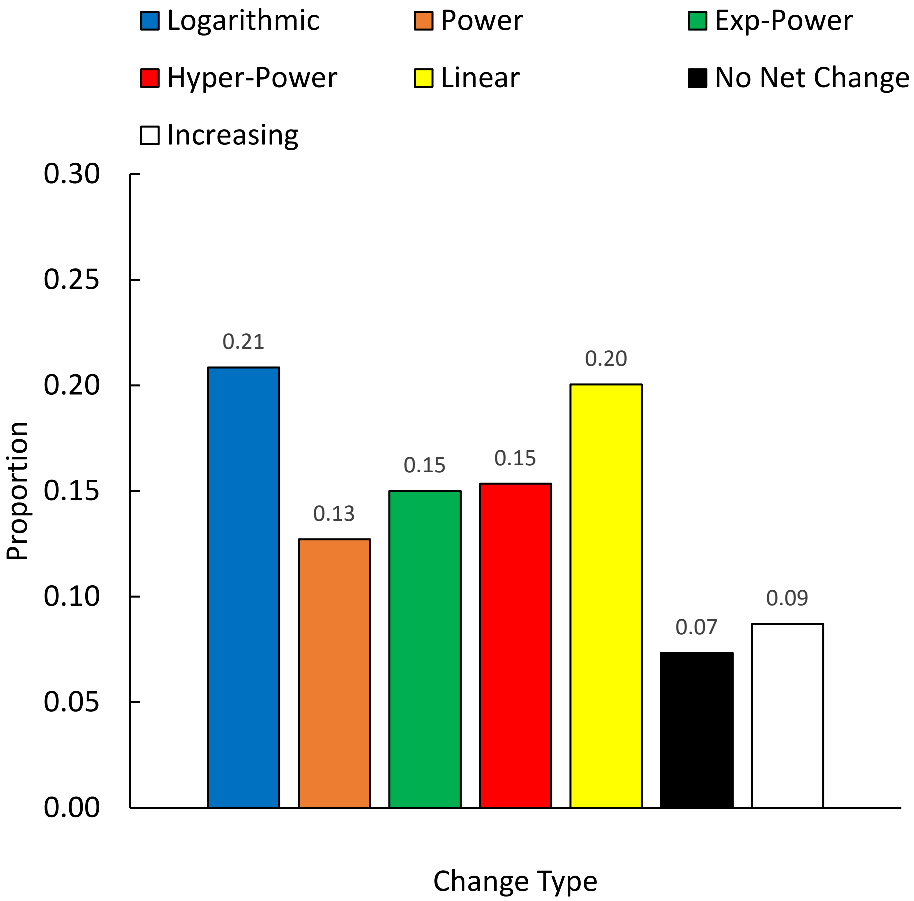

Assessment 3 (.90 or better, adjustment). This analysis is like the first in that it used the data sets that satisfied the criteria of fits of r^2 = .90 or better. However, here we weighted each data set based on the number of observations in each with the idea that studies that have relatively few observations should count less than studies with relatively large numbers of observations. As can be seen in Figure 3, the exponential-power function accounted for the largest proportion of data sets, followed by the linear function. The logarithmic and hyperbolic-power functions accounted for smaller proportions of data sets, and, again, the power function accounted for the least. This assessment also revealed a much larger contribution of no net change data sets.

Figure S3

The distribution of categories when only fits of .90, or were stable or improving data sets, are considered and there is an adjustment for the number of observations.

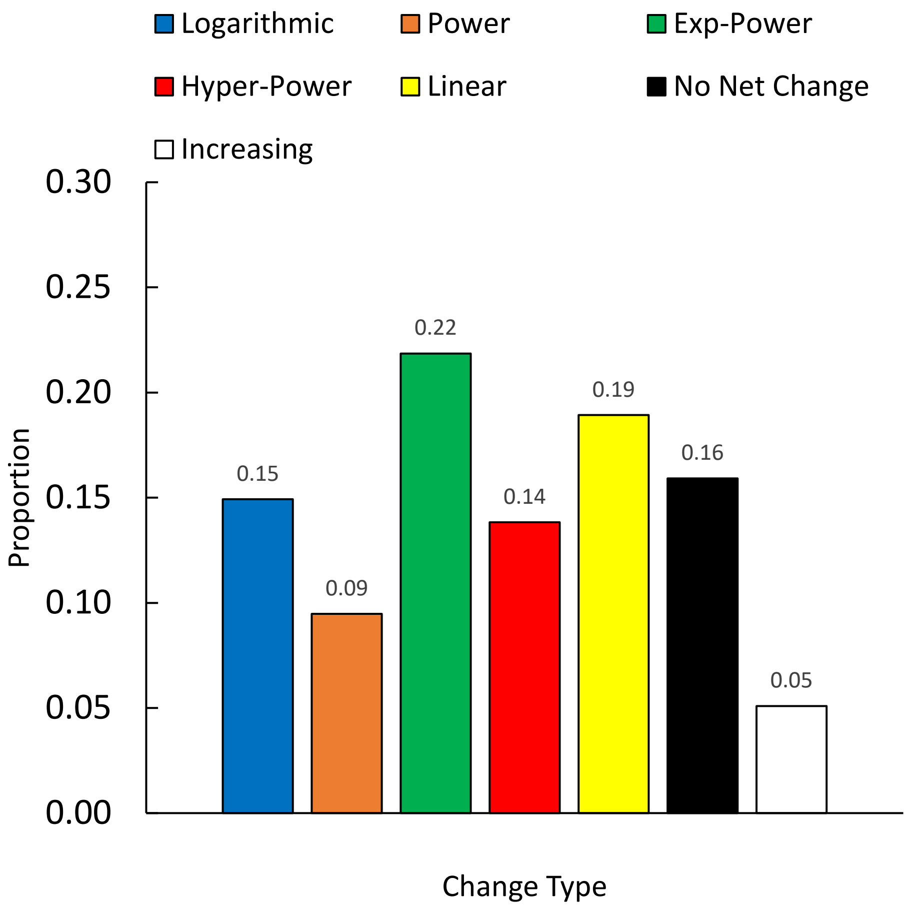
Logarithmic
Power
Exp-Power
Hyper-Power
Linear
No Net Change
Increasing
0.30
0.25
0.20
0.15
0.10
0.05
0.00
Proportion
0.15
0.09
0.22
0.14
0.19
0.16
0.05
Change Type
