# Supplementary material for: Memory from nonsense syllables to novels: A survey of retention
Source: Psychon Bull Rev. 2024 May 7;31(6):2437–64. doi: 10.3758/s13423-024-02514-3 (PMC11680664; doi:10.3758/s13423-024-02514-3)
Supplement: Supplementary file 2 — Supplementary file2 (PDF 143 KB) [file 13423_2024_2514_MOESM2_ESM.pdf]

Supplement D – Alternative Individual Factor and Regression Analyses

These are the individual factor analyses excluding those cases in which there was No net loss or increasing patterns.

Table D.1
Results of Tukey test comparisons for each of the characteristics. Those comparisons that did not reach significance are not shown.

Factor	Tukey test results	ANOVA
Year	Power < Linear (t = 3.32, p = .008, d = .41)	F = 2.94, p = .02, η_p^2 = .02
Complexity	Power < Linear (t = 5.78, p < .001, d = .72)<br>Power < Logarithmic (t = 3.23, p = .01, d = .40)<br>Power < Hyperbolic-power (t = 2.60, p < .07, d = .34)<br>Linear > Exponential-power (t = 3.59, p = .003, d = .42)<br>Linear > Hyperbolic-power (t = 3.29, p = .009, d = .38)<br>Linear > Logarithmic (t = 3.01, p = .02, d = .32)	F = 8.93, p < .001, η_p^2 = .05
Multiple Study?	Power < Linear (t = 4.89, p < .001, d = .61)<br>Power < Exponential-power (t = 3.70, p = .002, d = .49)<br>Power < Logarithmic (t = 2.87, p = .04, d = .35)<br>Linear > Hyperbolic-power (t = 2.96, p = .03, d = .34)	F = 6.79, p < .001, η_p^2 = .04
Distractor	Exponential-power > Linear (t = 3.62, p = .003, d = .42)	F = 3.39, p = .009, η_p^2 = .02
Free recall	None	F < 1
Cued recall	None	F = 1.07, p = .37, η_p^2 = .01
Recognition	Hyperbolic-power > Linear (t = 2.77, p = .05, d = .32)	F = 2.12, p =.08, η_p^2 = .01
Multiple Choice	Linear > Power (t = 4.14, p < .001, d = .51)<br>Linear > Exponential-power (t = 4.10, p = .001, d = .48)<br>Linear > Logarithmic (t = 3.77, p = .002, d = .40)<br>Linear > Hyperbolic-power (t = 2.45, p = .10, d = .28)	F = 6.54, p < .001, η_p^2 = .04
Savings	Power > Hyperbolic-power (t = 4.91, p < .001, d = .64)<br>Power > Linear (t = 5.16, p < .001, d = .64)<br>Power > Logarithmic (t = 4. 54, p < .001, d = .59)<br>Power > Exponential-power (t = 3.57, p = .004, d = .47)	F = 8.19, p < .001, η_p^2 = .04
Stem/Fragment Completion	Power > Hyperbolic-power (t = 2.97, p = .03, d = .38)<br>Power > Exponential-power (t =2.65, p = .06, d = .35)<br>Logarithmic > Hyperbolic-power (t = 3.01, p = .02, d = .34)<br>Logarithmic > Exponential-power (t =2.64, p = .06, d = .30)	F = 4.15, p = .002, η_p^2 = .02
Design	Logarithmic < Hyperbolic-power (t =2.87, p = .03, d = .33)<br>Logarithmic < Power (t =2.61, p = .07, d = .32)	F = 3.42, p = .009, η_p^2 = .02
Amount of data	None	F = 1.27, p = .28, η_p^2 = .01
Num. of RI	Hyperbolic-power < Logarithmic (t = 4.47, p < .001, d = .51)<br>Hyperbolic-power < Linear (t = 3.22, p = .01, d = .37)<br>Hyperbolic-power < Power (t = 3.08, p = .02, d = .40)	F = 5.61, p < .001, η_p^2 = .03
Longest RI	Hyperbolic-power < Logarithmic (t = 2.81, p = .04, d = .32)<br>Linear > Hyperbolic-power (t = 8.41, p < .001, d = .97)<br>Linear > Power (t = 7.80, p < .001, d = .97)<br>Linear > Exponential-power (t = 7.36, p < .001, d = .85)<br>Linear > Logarithmic (t = 6.08, p < .001, d = .65)	F = 25.59, p < .001, η_p^2 = .13
Initial memory	Exponential-power > Logarithmic (t = 3.89, p = .001, d = .45)<br>Exponential-power > Power (t = 3.40, p = .02, d = .45)	F = 4.88, p < .001, η_p^2 = .03

Table D.2
Results of logistic regressions for each of the characteristics.

	Logarithmic	Power	Exp-Power	Hyp-Power	Linear
Year					$p = .009$; $z = 2.63$
Complexity		$p = .03$; $z = -2.17$			
Multiple Study?		$p < .001$; $z = -3.61$			
Distractor			$p = .02$; $z = 2.36$		
Free Recall					
Cued Recall					
Recognition					
Multiple Choice					
Savings	$p = .07$; $z = 1.79$	$p = .08$; $z = 1.76$			
Completion					
Design	$p = .02$; $z = -2.33$			$p = .04$; $z = 2.09$	
Amount of data					
Num. of RI	$p < .001$; $z = 3.47$			$p = .01$; $z = -2.50$	
Longest RI			$p = .03$; $z = -2.22$	$p < .001$; $z = -3.23$	$p < .001$; $z = 5.32$
Initial memory			$p = .001$; $z = 3.29$		
