# Supplementary material for: Memory from nonsense syllables to novels: A survey of retention
Source: Psychon Bull Rev. 2024 May 7;31(6):2437–64. doi: 10.3758/s13423-024-02514-3 (PMC11680664; doi:10.3758/s13423-024-02514-3)
Supplement: Supplementary file 3 — Supplementary file3 (PDF 304 KB) [file 13423_2024_2514_MOESM3_ESM.pdf]

Supplement E – Decision Tree Framework

This model creates decision rules using the datasets we gathered to predict the function that will best fit the data for specific sets of variable values (included variables and levels of categorical variables are shown in Table E.1).

Table E.1

Variables included in the model.

Variables	Levels
Year	Numeric
Complexity	1, 2, 3, 4, 5, 6, 7 (refer to Table 5)
Studied > 1	0, 1
Memory Test	Cued Recall, Matching, Free Recall, Multiple Choice, Recognition, Completion, Savings, Problem Solving, Source Monitoring, Anagram Solution
Distractor	0, 1
Design	Within, Between
Data Amount	Numeric
Num RI's	Numeric
Longest RI	Numeric
Initial Memory	Numeric

Note. Only the levels shown here should be used when inputting characteristics for Complexity, Studied > 1, Memory Test, Distractor, and Design.

To train the model, we randomly selected 70% of datasets and then evaluated the model's performance on the remaining 30%. The model's overall testing accuracy was 31.27% and its accuracy for predicting each function is shown in Figures E.1. The testing precision and recall for each function are presented in Figure E.2 and a confusion matrix is shown in Table E.2.

Figure E.1

Testing accuracy for each function.

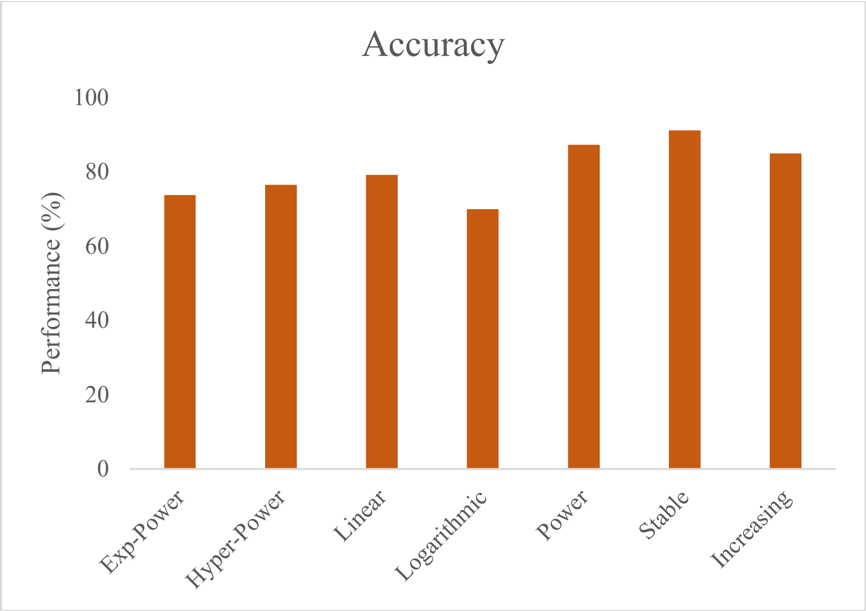

Figure E.2

Testing accuracy for each function.

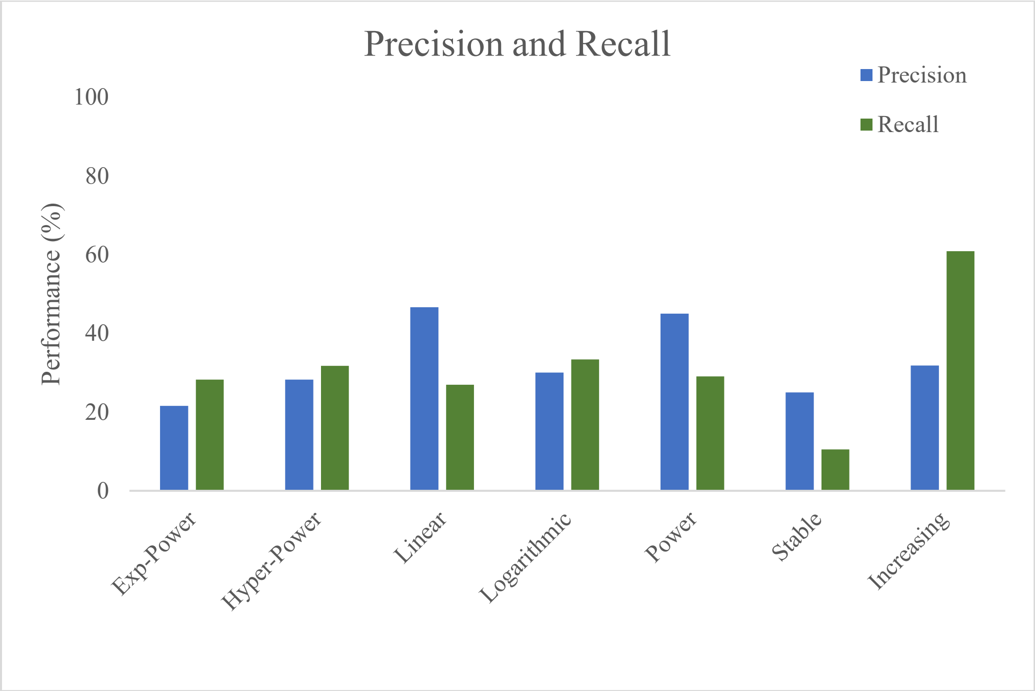

Table E.2

Model's confusion matrix.

	Predicted Exp-Power	Predicted Hyper-Power	Predicted Linear	Predicted Logarithmic	Predicted Power	Predicted Stable	Predicted Increasing
**True Exp-Power**	11	9	9	11	5	4	2
**True Hyper-Power**	6	13	10	9	5	2	1
**True Linear**	5	2	14	5	2	1	1
**True Logarithmic**	10	7	7	18	7	7	4
**True Power**	2	2	1	5	9	1	0
**True Stable**	0	2	1	1	1	2	1
**True Increasing**	5	6	10	5	2	2	14

The python code used for the model with code descriptions is shown below. The decision tree created by the model is also shown. In addition, we have added a jupyter file containing the code, along with a short video demonstrating how to input variables into the model to obtain its output.

Python code.

```
import pandas as pd
from C4point5 import C45
from chefboost import Chefboost as chef
from sklearn.model_selection import train_test_split

#opens document (path is unique for each computer)
df = pd.read_excel("C:\\Users\dparra2\Downloads\A Survey of Retention Working
Expanded Version (reduced).xlsx", index_col=0)

#reset index & delete last few rows that are empty
df.reset_index(inplace=True)
df = df.drop(index=df.index[-12:])

#including only relevant columns
df = df[['Year','Complexity', 'Studied > 1','Memory
test','Distractor','Design', ' Data Amount', "Num RI's", "Longest RI",
'Initial Memory','Type']]

df['Memory test'].replace(['Fragment Completion', 'stem completion', 'Stem
Completion','Stem completion', 'Fragment
completion'],['Completion','Completion','Completion','Completion','Completion
'], inplace=True)
df['Memory test'].replace(['savings','Savings'],['Savings','Savings'],
inplace=True)
df['Design'].replace(['within','between'],['Within','Between'], inplace=True)

#displays dataframe
pd.set_option('display.max_columns', None)
```

```
df.head()

#train-test split
df = df.rename(columns={"Type":"Decision"})
X_train, X_test = train_test_split(df, test_size=0.3, random_state=22,
stratify=df["Decision"])

#specify configuration (from: https://github.com/serengil/chefboost)
config = {'algorithm': 'C4.5'}

#build decision tree
model = chef.fit(X_train, config = config)
evaluation = chef.evaluate(model, X_test, task="test")
def prediction(Year, Complexity, Studied, Memory_Test, Distractor, Design,
DataAmt, NumRI, Longest, InitialMem):
    moduleName = "outputs/rules/rules" #this will load outputs/rules/rules.py
    tree = chef.restoreTree(moduleName)
    pred = tree.findDecision([Year, Complexity, Studied, Memory_Test,
Distractor, Design, DataAmt, NumRI, Longest, InitialMem])
    print(pred)

#asks for input
Year =int(input("Year?"))
Complexity = int(input("Complexity?"))
Studied = int(input("Studied>1?"))
Memory_Test = input("Memory Test?")
Distractor = int(input("Distractor?"))
Design = input("Design?")
DataAmt = int(input("Data Amount?"))
NumRI = int(input("Number of Retention Intervals?"))
Longest = int(input("Longest Retention Interval (seconds)?"))
InitialMem = float(input("Initial Memory Score? "))

prediction(Year, Complexity, Studied, Memory_Test, Distractor, Design,
DataAmt, NumRI, Longest, InitialMem)
```

Decision Tree.

```
def findDecision(obj): #obj[0]: Year, obj[1]: Complexity, obj[2]: Studied >
1, obj[3]: Memory test, obj[4]: Distractor, obj[5]: Design, obj[6]:  Data
Amount, obj[7]: Num RI's, obj[8]: Longest RI, obj[9]: Initial Memory
    # {"feature": "Initial Memory", "instances": 604, "metric_value": 2.7224,
"depth": 1}
    if obj[9]>0.027:
        # {"feature": "Longest RI", "instances": 603, "metric_value": 2.7211,
"depth": 2}
        if obj[8]>5.9:
            # {"feature": "Year", "instances": 602, "metric_value": 2.7205,
"depth": 3}
            if obj[0]>1907.0:
                # {"feature": "Num RI's", "instances": 601, "metric_value":
2.7205, "depth": 4}
                if obj[7]<=12.0:
                    # {"feature": " Data Amount", "instances": 599,
"metric_value": 2.7221, "depth": 5}
                    if obj[6]>10.0:
```

```
                        # {"feature": "Complexity", "instances": 597,
"metric_value": 2.7216, "depth": 6}
                        if obj[1]>2.0:
                            # {"feature": "Distractor", "instances": 341,
"metric_value": 2.6878, "depth": 7}
                            if obj[4]<=0.0:
                                # {"feature": "Studied > 1", "instances":
299, "metric_value": 2.68, "depth": 8}
                                if obj[2]>0.0:
                                    # {"feature": "Design", "instances": 173,
"metric_value": 2.531, "depth": 9}
                                    if obj[5] == 'Within':
                                        # {"feature": "Memory test",
"instances": 124, "metric_value": 2.3981, "depth": 10}
                                        if obj[3] == 'Free Recall':
                                            return 'Increasing'
                                        elif obj[3] == 'Multiple Choice':
                                            return 'Linear'
                                        elif obj[3] == 'Recognition':
                                            return 'Logarithmic'
                                        elif obj[3] == 'Cued Recall':
                                            return 'Increasing'
                                        else: return 'Increasing'
                                    elif obj[5] == 'Between':
                                        # {"feature": "Memory test",
"instances": 49, "metric_value": 2.3665, "depth": 10}
                                        if obj[3] == 'Free Recall':
                                            return 'Logarithmic'
                                        elif obj[3] == 'Cued Recall':
                                            return 'Exp-Power'
                                        elif obj[3] == 'Multiple Choice':
                                            return 'Logarithmic'
                                        elif obj[3] == 'Recognition':
                                            return 'Linear'
                                        elif obj[3] == 'Problem Solving':
                                            return 'Linear'
                                        else: return 'Linear'
                                    else: return 'Logarithmic'
                                elif obj[2]<=0.0:
                                    # {"feature": "Design", "instances": 126,
"metric_value": 2.6195, "depth": 9}
                                    if obj[5] == 'Within':
                                        # {"feature": "Memory test",
"instances": 68, "metric_value": 2.3119, "depth": 10}
                                        if obj[3] == 'Free Recall':
                                            return 'Hyper-Power'
                                        elif obj[3] == 'Multiple Choice':
                                            return 'Hyper-Power'
                                        elif obj[3] == 'Cued Recall':
                                            return 'Logarithmic'
                                        elif obj[3] == 'Recognition':
                                            return 'Hyper-Power'
                                        else: return 'Hyper-Power'
                                    elif obj[5] == 'Between':
                                        # {"feature": "Memory test",
"instances": 58, "metric_value": 2.6853, "depth": 10}
                                        if obj[3] == 'Free Recall':
```

```
                                        return 'Logarithmic'
                                    elif obj[3] == 'Recognition':
                                        return 'Logarithmic'
                                    elif obj[3] == 'Multiple Choice':
                                        return 'Logarithmic'
                                    elif obj[3] == 'Cued Recall':
                                        return 'Exp-Power'
                                    elif obj[3] == 'Source Monitoring':
                                        return 'Logarithmic'
                                    else: return 'Logarithmic'
                                else: return 'Logarithmic'
                            else: return 'Hyper-Power'
                        elif obj[4]>0.0:
                            # {"feature": "Memory test", "instances": 42,
"metric_value": 2.3458, "depth": 8}
                            if obj[3] == 'Cued Recall':
                                # {"feature": "Studied > 1", "instances":
22, "metric_value": 2.0543, "depth": 9}
                                if obj[2]>0.0:
                                    # {"feature": "Design", "instances":
17, "metric_value": 1.9831, "depth": 10}
                                    if obj[5] == 'Within':
                                        return 'Exp-Power'
                                    elif obj[5] == 'Between':
                                        return 'Logarithmic'
                                    else: return 'Logarithmic'
                                elif obj[2]<=0.0:
                                    # {"feature": "Design", "instances":
5, "metric_value": 1.9219, "depth": 10}
                                    if obj[5] == 'Between':
                                        return 'Exp-Power'
                                    elif obj[5] == 'Within':
                                        return 'Hyper-Power'
                                    else: return 'Hyper-Power'
                                else: return 'Hyper-Power'
                            elif obj[3] == 'Free Recall':
                                # {"feature": "Design", "instances": 17,
"metric_value": 2.3243, "depth": 9}
                                if obj[5] == 'Within':
                                    # {"feature": "Studied > 1",
"instances": 9, "metric_value": 2.5033, "depth": 10}
                                    if obj[2]<=0.0:
                                        return 'Exp-Power'
                                    else: return 'Exp-Power'
                                elif obj[5] == 'Between':
                                    # {"feature": "Studied > 1",
"instances": 8, "metric_value": 1.8113, "depth": 10}
                                    if obj[2]>0.0:
                                        return 'Exp-Power'
                                    elif obj[2]<=0.0:
                                        return 'Logarithmic'
                                    else: return 'Logarithmic'
                                else: return 'Logarithmic'
                            elif obj[3] == 'Recognition':
                                return 'Exp-Power'
                            elif obj[3] == 'Matching':
                                return 'Power'
```

```
                    else: return 'Power'
                else: return 'Logarithmic'
            elif obj[1]<=2.0:
                # {"feature": "Memory test", "instances": 256,
"metric_value": 2.5888, "depth": 7}
                if obj[3] == 'Free Recall':
                    # {"feature": "Design", "instances": 100,
"metric_value": 2.5116, "depth": 8}
                    if obj[5] == 'Within':
                        # {"feature": "Studied > 1", "instances":
84, "metric_value": 2.4775, "depth": 9}
                        if obj[2]<=0.0:
                            # {"feature": "Distractor",
"instances": 57, "metric_value": 2.4976, "depth": 10}
                            if obj[4]>0.0:
                                return 'Exp-Power'
                            elif obj[4]<=0.0:
                                return 'Linear'
                            else: return 'Linear'
                        elif obj[2]>0.0:
                            # {"feature": "Distractor",
"instances": 27, "metric_value": 2.1337, "depth": 10}
                            if obj[4]<=0.0:
                                return 'Exp-Power'
                            elif obj[4]>0.0:
                                return 'Exp-Power'
                            else: return 'Exp-Power'
                        else: return 'Exp-Power'
                    elif obj[5] == 'Between':
                        # {"feature": "Distractor", "instances":
16, "metric_value": 2.2806, "depth": 9}
                        if obj[4]<=0.0:
                            # {"feature": "Studied > 1",
"instances": 13, "metric_value": 2.0382, "depth": 10}
                            if obj[2]<=0.0:
                                return 'Logarithmic'
                            elif obj[2]>0.0:
                                return 'Exp-Power'
                            else: return 'Exp-Power'
                        elif obj[4]>0.0:
                            # {"feature": "Studied > 1",
"instances": 3, "metric_value": 1.585, "depth": 10}
                            if obj[2]<=0.0:
                                return 'Increasing'
                            else: return 'Increasing'
                        else: return 'Increasing'
                    else: return 'Logarithmic'
                elif obj[3] == 'Recognition':
                    # {"feature": "Design", "instances": 69,
"metric_value": 2.6443, "depth": 8}
                    if obj[5] == 'Within':
                        # {"feature": "Distractor", "instances":
41, "metric_value": 2.3183, "depth": 9}
                        if obj[4]<=0.0:
                            # {"feature": "Studied > 1",
"instances": 37, "metric_value": 2.23, "depth": 10}
                            if obj[2]<=0.0:
```

```
                                    return 'Hyper-Power'
                                elif obj[2]>0.0:
                                    return 'Hyper-Power'
                                else: return 'Hyper-Power'
                            elif obj[4]>0.0:
                                # {"feature": "Studied > 1",
"instances": 4, "metric_value": 2.0, "depth": 10}
                                if obj[2]<=0.0:
                                    return 'Power'
                                else: return 'Power'
                            else: return 'Power'
                        elif obj[5] == 'Between':
                            # {"feature": "Studied > 1", "instances":
28, "metric_value": 2.629, "depth": 9}
                            if obj[2]<=0.0:
                                # {"feature": "Distractor",
"instances": 22, "metric_value": 2.5561, "depth": 10}
                                if obj[4]<=0.0:
                                    return 'Stable'
                                elif obj[4]>0.0:
                                    return 'Power'
                                else: return 'Power'
                            elif obj[2]>0.0:
                                # {"feature": "Distractor",
"instances": 6, "metric_value": 1.2516, "depth": 10}
                                if obj[4]<=0.0:
                                    return 'Linear'
                                else: return 'Linear'
                            else: return 'Linear'
                        else: return 'Stable'
                    elif obj[3] == 'Multiple Choice':
                        # {"feature": "Distractor", "instances": 33,
"metric_value": 2.4434, "depth": 8}
                        if obj[4]<=0.0:
                            # {"feature": "Studied > 1", "instances":
31, "metric_value": 2.3835, "depth": 9}
                            if obj[2]<=0.0:
                                # {"feature": "Design", "instances":
20, "metric_value": 2.3282, "depth": 10}
                                if obj[5] == 'Within':
                                    return 'Hyper-Power'
                                elif obj[5] == 'Between':
                                    return 'Logarithmic'
                                else: return 'Logarithmic'
                            elif obj[2]>0.0:
                                # {"feature": "Design", "instances":
11, "metric_value": 1.9808, "depth": 10}
                                if obj[5] == 'Within':
                                    return 'Exp-Power'
                                elif obj[5] == 'Between':
                                    return 'Hyper-Power'
                                else: return 'Hyper-Power'
                            else: return 'Exp-Power'
                        elif obj[4]>0.0:
                            # {"feature": "Studied > 1", "instances":
2, "metric_value": 1.0, "depth": 9}
                            if obj[2]<=0.0:
```

```
                                            # {"feature": "Design", "instances":
2, "metric_value": 1.0, "depth": 10}
                                            if obj[5] == 'Between':
                                                return 'Linear'
                                            else: return 'Linear'
                                        else: return 'Linear'
                                    else: return 'Linear'
                                elif obj[3] == 'Completion':
                                    # {"feature": "Distractor", "instances": 23,
"metric_value": 2.0018, "depth": 8}
                                    if obj[4]<=0.0:
                                        # {"feature": "Design", "instances": 19,
"metric_value": 1.74, "depth": 9}
                                        if obj[5] == 'Within':
                                            # {"feature": "Studied > 1",
"instances": 18, "metric_value": 1.7642, "depth": 10}
                                            if obj[2]<=0.0:
                                                return 'Power'
                                            elif obj[2]>0.0:
                                                return 'Logarithmic'
                                            else: return 'Logarithmic'
                                        elif obj[5] == 'Between':
                                            return 'Logarithmic'
                                        else: return 'Logarithmic'
                                    elif obj[4]>0.0:
                                        # {"feature": "Studied > 1", "instances":
4, "metric_value": 1.5, "depth": 9}
                                        if obj[2]<=1.0:
                                            # {"feature": "Design", "instances":
4, "metric_value": 1.5, "depth": 10}
                                            if obj[5] == 'Between':
                                                return 'Linear'
                                            else: return 'Linear'
                                        else: return 'Linear'
                                    else: return 'Linear'
                                elif obj[3] == 'Cued Recall':
                                    # {"feature": "Design", "instances": 22,
"metric_value": 2.1506, "depth": 8}
                                    if obj[5] == 'Between':
                                        # {"feature": "Distractor", "instances":
11, "metric_value": 1.9717, "depth": 9}
                                        if obj[4]<=0.0:
                                            # {"feature": "Studied > 1",
"instances": 9, "metric_value": 1.7527, "depth": 10}
                                            if obj[2]>0.0:
                                                return 'Hyper-Power'
                                            elif obj[2]<=0.0:
                                                return 'Hyper-Power'
                                            else: return 'Hyper-Power'
                                        elif obj[4]>0.0:
                                            # {"feature": "Studied > 1",
"instances": 2, "metric_value": 1.0, "depth": 10}
                                            if obj[2]>0.0:
                                                return 'Linear'
                                            elif obj[2]<=0.0:
                                                return 'Hyper-Power'
                                            else: return 'Hyper-Power'
```

```
                                    else: return 'Linear'
                                elif obj[5] == 'Within':
                                    # {"feature": "Studied > 1", "instances": 
10, "metric_value": 0.469, "depth": 9}
                                    if obj[2]<=0.0:
                                        # {"feature": "Distractor", 
"instances": 10, "metric_value": 0.469, "depth": 10}
                                        if obj[4]<=0.0:
                                            return 'Power'
                                        else: return 'Power'
                                    else: return 'Power'
                                else: return 'Power'
                            elif obj[3] == 'Savings':
                                # {"feature": "Design", "instances": 6, 
"metric_value": 1.2516, "depth": 8}
                                if obj[5] == 'Within':
                                    # {"feature": "Studied > 1", "instances": 
4, "metric_value": 1.5, "depth": 9}
                                    if obj[2]<=1.0:
                                        # {"feature": "Distractor", 
"instances": 4, "metric_value": 1.5, "depth": 10}
                                        if obj[4]<=0.0:
                                            return 'Power'
                                        else: return 'Power'
                                    else: return 'Power'
                                elif obj[5] == 'Between':
                                    return 'Power'
                                else: return 'Power'
                            elif obj[3] == 'Matching':
                                return 'Exp-Power'
                            elif obj[3] == 'Anagram solution':
                                return 'Logarithmic'
                            else: return 'Logarithmic'
                        else: return 'Logarithmic'
                    elif obj[6]<=10.0:
                        # {"feature": "Memory test", "instances": 2, 
"metric_value": 1.0, "depth": 6}
                        if obj[3] == 'Free Recall':
                            return 'Power'
                        elif obj[3] == 'Cued Recall':
                            return 'Hyper-Power'
                        else: return 'Hyper-Power'
                    else: return 'Power'
                elif obj[7]>12.0:
                    return 'Logarithmic'
                else: return 'Logarithmic'
            elif obj[0]<=1907.0:
                return 'Exp-Power'
            else: return 'Exp-Power'
        elif obj[8]<=5.9:
            return 'Power'
        else: return 'Power'
    elif obj[9]<=0.027:
        return 'Increasing'
    else: return 'Increasing'
```
